# De Novo Hybrid Assembled Draft Genome of *Commiphora wightii* (Arnott) Bhandari Reveals Key Enzymes Involved in Phytosterol Biosynthesis

**DOI:** 10.3390/life13030662

**Published:** 2023-02-28

**Authors:** Rudra Prasad Banerjee, Gopal Ji Tiwari, Babita Joshi, Satya Narayan Jena, Om Prakash Sidhu, Baleshwar Meena, Tikam S. Rana, Saroj K. Barik

**Affiliations:** 1 CSIR-National Botanical Research Institute, Rana Pratap Marg, Lucknow 226001, India; 2 Academy of Scientific and Innovative Research (AcSIR), Ghaziabad 201002, India

**Keywords:** phytosterol biosynthesis pathway, guggulsterone, flow cytometry-based genome size estimation, draft genome

## Abstract

Genome sequence and identification of specific genes involved in the targeted secondary metabolite biosynthesis are two essential requirements for the improvement of any medicinal plant. *Commiphora wightii* (Arnott) Bhandari (family: Burseraceae), a medicinal plant native to Western India, produces a phytosterol guggulsterone, which is useful for treating atherosclerosis, arthritis, high cholesterol, acne, and obesity. For enhanced guggulsterone yield, key genes involved in its biosynthesis pathway need to be predicted, for which the genome sequence of the species is a pre-requisite. Therefore, we assembled the first-ever hybrid draft genome of *C. wightii* with a genome size of 1.03 Gb and 107,221 contigs using Illumina and PacBio platforms. The N50 and L50 values in this assembled genome were ~74 Kb and 3486 bp, respectively with a guanine–cytosine (GC) content of 35.6% and 98.7%. The Benchmarking Universal Single Copy Ortholog (BUSCO) value indicated good integrity of assembly. Analysis predicted the presence of 31,187 genes and 342.35 Mb repeat elements in the genome. The comparative genome analysis of *C. wightii* with relevant orthogroups predicted a few key genes associated with phytosterol biosynthesis and secondary metabolism pathways. The assembled draft genome and the predicted genes should help the future variety development program with improved guggulsterone contents in *C. wightii*.

## 1. Introduction

*Commiphora wightii* (Arnott) Bhandari (family: Burseraceae) is a medicinally important plant, and is native to arid regions of Western India. It is also found in Bangladesh, Pakistan, China and tropical Africa [1]. Guggulsterone, a phytosterol derived from the resin of *C. wightii*, is being used in *Ayurveda* to treat obesity, arthritis, and hyperlipidemia [2,3]. It has also been shown to have nutraceutical, anti-arthritic, anti-inflammatory and anti-lipid properties [4]. Moreover, it also has an anticancer effect on a range of human tumor cell types [5]. The unsustainable extraction of resin from trees has resulted in a loss of its natural populations, and the species is now critically endangered [6,7].

Although many studies are available on the chemical characterization and medicinal importance of guggulsterone [3,6,8,9,10], no study has been carried out elucidating the regulation mechanism(s) of guggulsterone biosynthesis. As of today, even the genome sequence of the species is not available. Because of low phytosterol yield, a large number of trees are damaged every year due to unsustainable harvest leading to the drastic depletion of its natural populations. Therefore, one of the strategies for the conservation of *C. wightii* is to increase the production of guggulsterone using genomic approaches [11]. Genome sequencing is fundamental to such approaches as it helps in identifying the key signatures governing the important traits.

Next generation sequencing (NGS) is a powerful tool to understand the structural organization and function(s) of genes as well as pathways associated with guggulsterone biosynthesis. The hybrid assembly approach with a large amount of sequencing data for greater coverage and better chromosome-scale scaffolding with optical mapping or HiC has been proved to be effective for genome analysis. The hybrid approach to genome assembly uses cutting-edge technologies to combine short and long reads [12]. The amalgamation of these two sequencing approaches unravels the underlying structural genomics for specific traits [13,14]. Where no genomic data are available, draft genome with lesser sequencing data and scaffolding could be extremely useful [15,16]. Several trait-specific potential genes and gene families from different crops have previously been successfully mined and functionally validated using NGS-based draft and finished genomes along with other applications [17,18,19,20,21,22,23,24,25,26]. Plant metabolite enhancement programs have immensely benefited from the association data between the genome-wide scan and targeted alkaloid contents [27]. However, plants with a huge genome size and high levels of repetitiveness pose challenges in obtaining high quality sequences [28].

In the present study, a hybrid draft genome of *C. wightii*, commonly known as guggul with chromosome 2n = 2x = 26 [29], was assembled. An attempt was made to predict a few key genes involved in secondary metabolism and phytosterol biosynthesis pathways using the draft genome. The findings of the study should help in improving *C. wightii* for enhanced guggulsterone production.

## 2. Materials and Methods

### 2.1. Selection of Plant Materials for Genome Sequencing

CSIR-National Botanical Research Institute, Lucknow, India has a total of 53 guggul germplasms, which were collected from the state of Rajasthan, India (Indigenous collection numbers: IC-471198 to IC-471282). The young leaves from two-month-old seedlings of the genotype with the highest guggulsterone content of 20.37 mg g^−1^ (IC471203) were used for de novo draft genome sequencing.

### 2.2. Genomic DNA Isolation

The young leaf tissues were utilized for genomic DNA extraction using nuclei isolation buffer following a combination of several methods and commercial kits (Circulomics Inc., Baltimore, MD, USA) to obtain high molecular weight (HMW) DNA. Prior to sequencing, intactness of DNA was further confirmed by pulse field gel electrophoresis. The isolated genomic DNA was quantified following both QubitdsDNA HS assay (Invitrogen, Waltham, MA, USA) with QubitFluorometer, and colorimetric assay with Nanodrop (Nanodrop, Waltham, MA, USA).

### 2.3. Library Preparation and High-Throughput Sequencing

The genome sequencing was performed on both short read (NextSeq 550, Illumina Inc., San Diego, CA, USA) and long read (PacBio Sequel System, San Diego, CA, USA) platforms. NextSeq 550 sequencer was used to perform the paired end shotgun sequencing. The standard protocol of the NEBNext Ultra DNA Library Prep Kit (Illumina, USA) was used to construct the paired-end sequencing libraries with 300 bp insert lengths. Clonal clusters of each library were generated using cBot with TruSeq PE Cluster Kit v. 3-cBot-HS (Illumina) and sequenced using Hiseq X10 sequencer with a TrueSeq SBS Kit v.2-HS (Illumina, USA) by the pair end sequencing method for 150bp lengths. Two forms of software, namely Hiseq Control Software (HCS) v.2.0.12 and Real Time Analysis (RTA) v.1.17.21.3 (Illumina, USA), were used to analyze the sequence data. The raw reads generated after the sequencing were further pre-processed by trimming the adapter sequences based on quality using default parameters of the FASTX-Toolkit for fastqs data.

A library for the PacBio sequencing was constructed in the same manner using the SMRTBell Template Prep Kit 1.0 (Pacific Bioscience, Menlo Park, CA, USA) according to the manufacturer’s instructions. The sequencing reactions were performed in the SMRT cells by using the SMRTBell template sequencing primer with DNA polymerase. The reaction was run by maintaining the PacBio Sequel in a circular consensus sequencing (CCS) mode using the sequel DNA Polymerase 2.0 with new chemistry 2 (Sequel Binding Kit 2.0, Sequel Sequencing Kit 2.1). The sequencing data were analyzed using SMRT Analysis V.2.2.0 (Pacific biosciences). To estimate the genome size based on the sequencing reads, the *K*-mer frequency (*K*-value) was calculated using the *K*-mer Analysis Toolkit (KAT).

### 2.4. Flow Cytometry-Based Genome Size Estimation

The genome size of guggul was estimated in a flow cytometer (Cube 8, Konstanz, Germany, Europe) using the *Solanum lycopersicum* genome as reference. We selected *S. lycopersicum* as a reference standard because its peak overlapped with that of *C. wightii* when both the samples were run in the flow cytometer, suggesting closeness in their genome size. Young leaves from the apical meristem were used for sample preparation. Fresh leaves were chopped with a razor blade in ice cold nuclei isolation buffer (Cystain 3 Absolute) and filtered through 35µL mesh. The whole nuclei suspension was added with PI staining solution and incubated for 1 h in dark [30]. After 1 h, the resulted nuclei suspension with labelled DNA was subjected to flow cytometer (Cube 8, Germany, Europe) for measuring relative DNA content. The histogram peaks were used to measure the mean 2C DNA content of collected guggul samples. The median position of G0/G1 peaks in the histograms of the guggul sample was statistically corrected by taking the mean value of the medians with three replicates. The haploid genome size of guggul was estimated by comparing that with 950 Mb *Solanum lycopercum* genome with 2C = 1.96 pg [31,32,33] following the formula mentioned below:

$$\mathrm{Sample}\;2C\;\mathrm{DNA}\;\left(\mathrm{pg}\right)=\frac{\mathrm{Mean}\;\mathrm{of}\;\mathrm{sample}\;2C\;\mathrm{peak}}{\mathrm{Mean}\;\mathrm{of}\;\mathrm{standard}\;2C\;\mathrm{peak}}\;\mathrm{standard}\;2C\;\mathrm{value}$$

### 2.5. Genome Assembly and Evaluation of Assembled Genome

Several methods were adopted for individual read assembly as well as hybrid genome assembly. To perform the assembly of the sequences, the fastq files were preprocessed at the beginning. Adapter sequences were trimmed and filtered out. Only the reads with average quality score >20 were retained and processed further for downstream analysis. De novo assembly was performed in MaSuRCA [34] by using both PacBio and Illumina cleaned reads with an optimized *k*-mer length of 48. MaSuRCA assembly was used for further downstream analyses to obtain better statistical coverage. We tried optimization with the Canu for better assembly. However, we could not achieve any substantial improvement with the Canu assembler tool as compared to MaSuRCA. Even the N50 value of MaSuRCA was similar to that of Canu. Therefore, we used only MaSuRCA as our optimized assembly tool. Two methods, viz., Benchmarking Universal Single-Copy Orthologs (BUSCO) and the Quality Assessment Tool for genome assemblies (QUAST) were used to evaluate the quality of the final assembly. The BUSCO v5.4.2 approach was used to evaluate the accuracy and completeness of the genome assembly while QUAST 4.0 was used to determine the completeness of Illumina paired end reads that were mapped during the final assembly with the default parameters [35]. BUSCO built several large single copy gene sets based on the OrthoDB database with evolutionary tree. The BUSCO assessment was performed using Viridaeplantae, Eudicots and Embryophyta plant lineage datasets for a total of 138 genomes (57 genomes for Viridaeplantae, 31 genomes for Eudicots and 50 genomes for Embryophyta). The BUSCO was executed in genome mode considering all the conserved orthologs available in the Viridaeplantae, Eudicots and Embryophyta lineages. A total of 4365 conserved orthologous genes (425 in Viridaeplantae, 2326 in Eudicotsand and 1614 in Embryophyta) were considered for the evaluation of genome.

### 2.6. Analysis of Repetitive Elements

The de novo repetitive sequences in *C. wightii* genome were identified using RepeatModeler (v.1.0.8) based on a self-blast search. In addition, known repetitive sequences were searched through RepeatMasker (v 2.2) using a cross-match program with a Repbase-derived RepeatMasker library. The de novo repetitive sequences were further constructed using RepeatModeler (v20170127).

### 2.7. Gene Prediction and Functional Annotation

The MaSuRCA assembled contigs were used to predict the coding sequences (CDSs) through Augustus 3.2.3. The in-house Perl script was used to annotate the predicted genes with the following three steps: (i) matching with the UniProt database using the BLASTX program, (ii) organism annotation and (iii) gene ontology. At first, the predicted genes were compared with the UniProt database using the BLASTX program with an E-value cutoff of 10-3. The best BLASTX hits were filtered based on their identity, query coverage and similarity score. Further, the description of each gene was filtered out using our in-house pipeline. In the second step, the top BLASTX hit was also searched for each gene to identify the top 10 organisms. Lastly, the gene ontology (GO) in terms of molecular function (MF), cellular component (CC) and biological process (BP) was mapped using a pipeline developed in house for such categorization.

The protein sequences were analyzed using a KEGG automation annotation server (KAAS). All the pathways were further screened to understand the enzymes involved in phytosterol biosynthesis and important secondary metabolism. The gene sequences were aligned to the phytosterol genes, downloaded from UniProt andfollowed by filtration based on top hits.

### 2.8. Phylogenetics and Synteny Analysis

Phylogenetic analysis and synteny plot were obtained using Mega version 7.0. Orthologous sequences were identified with the help of BUSCO and Orthofinder tools for the 23 plant genomes available in the public database along with guggul samples. The integrated supergene sequence was constructed based on the four-fold degenerated sites (4DTv sites) of the orthogroups. The orthogroups obtained by Orthofinder were used to predict the phylogenetic relationship among the different plant species. Mega version 7.0 was used to predict the phylogenetic tree for all the genomes. The maximum parsimony algorithm for phylogenetic analysis with a bootstrap value of 1000 was used. However, due to the unavailability of any sequence data of the other members of the family Burseraceae, the genome of *C. wightii* was compared with other closely related species. Therefore, additional searches were carried out to find out the reference genome sequence across other closely allied orders and families such as Rutaceae, Brassicaceae, Malvaceae, Myrtaceae and Lythraceae. The genome of *Citrus sinensis* was fetched and organized as a database for the homology search. Each contig from the genome of interest was subjected to a homology search against *Citrus sinensis* database with cut-off query coverage length of one kb. The matching hit contigs of *C. weightii* for each chromosome of *C. sinensis* were fetched for preparing the synteny map and visualized in a Circos plot as total percentage of collinearity. The position of all collinear contigs and their coverage was calculated for each chromosome of *C. sinensis* and converted into % of homology. BLASTP V2.2.31 was used to analyze the aligned protein sequences of *C. wightii* and *Citrus sinensis* (Rutaceae). Circos was used to visualize these genomic synteny block data (http://circos.ca/software, accessed on 20 January 2023).

### 2.9. Development of SSR Genetic Marker Resources

The Perl script MIcroSAtelitte (MISA) was used to identify microsatellites in the *C. wightii* genome. The length of nucleotide motifs to identify SSRs was kept from 2 to 6 and the minimum repeat unit was defined as 6 for di-nucleotides, 5 for tri-nucleotides, 4 for tetra-nucleotides, and 3 for penta and hexa-nucleotides. The compound SSRs were categorized by the presence of more than 100 bp in between two SSRs. The designing of primers flanking the repeat regions was carried out by obtaining the SSR information generated by MISA. Two perl scripts were used as interface modules for data interchange between MISA and the primer 3. Only those regions that contained sufficient sequences on both flanking sides of SSR repeats were selected for the development of SSR markers. The designing of SSR primers was carried out by considering the parameters such as primer length (18–24 bp with 20 bp as the optimum), percentage of GC (40–70% with optimum value of 50%), primer Tm (50–60 °C) and product size range (100–500 bp).

### 2.10. Data Availability

The data generated after assembly were submitted to the NCBI open-access database for use in academic and commercial purposes (SRA Accession Number for Ilumina- SRR12931174 and PacBio- SRR12931173, SRR12931172, and SRR12931171; Bio-project accession number: PRJNA645081; BioSample accession number: SAMN15491827). The data can be accessed by following the link: https://dataview.ncbi.nlm.nih.gov/object/PRJNA645081?reviewer=kmjuh8jqst2446f2bc990r6d9k. All the Supplemental Files and related figures and tables are uploaded in a separate format. The legends of all the figures, tables and Supplemental Material are provided at the end of the manuscript.

## 3. Results

### 3.1. Obtaining HMW Genomic DNA and Sequencing

The quantified value of the isolated DNA was 256 ng/µL with a ratio of 1.84 (260/280). Results obtained from pulse field gel electrophoresis were used to select the larger DNA fragments with proper intactness. PacBio-based long read sequencing in SMRT cells generated a total of ~32 Gb of raw data (Supplementary Figures S1 and S3, Table 1). The raw data for the SMRT cells varied from 10.6 GB to 11.28 GB with a varied number of polymerase reads from 811,704 (mean read length of 13,904 bp and 24,250 bp of polymerase read N50) to 868,985 (mean read length of 12,781 bp and 23,750 bp of polymerase read N50). Subsequently, pair-end sequencing through the Illumina platform was generated for a total of ~124 Gb data. A total number of 827,455,092 paired-end reads with 124,118.26 Gb data and with 43.61% GC content were generated and performed for quality score (Supplementary Figure S2, Table 1).

### 3.2. Flow Cytometry (FCM)-Based Genome Size Estimation

**Figure 1:**
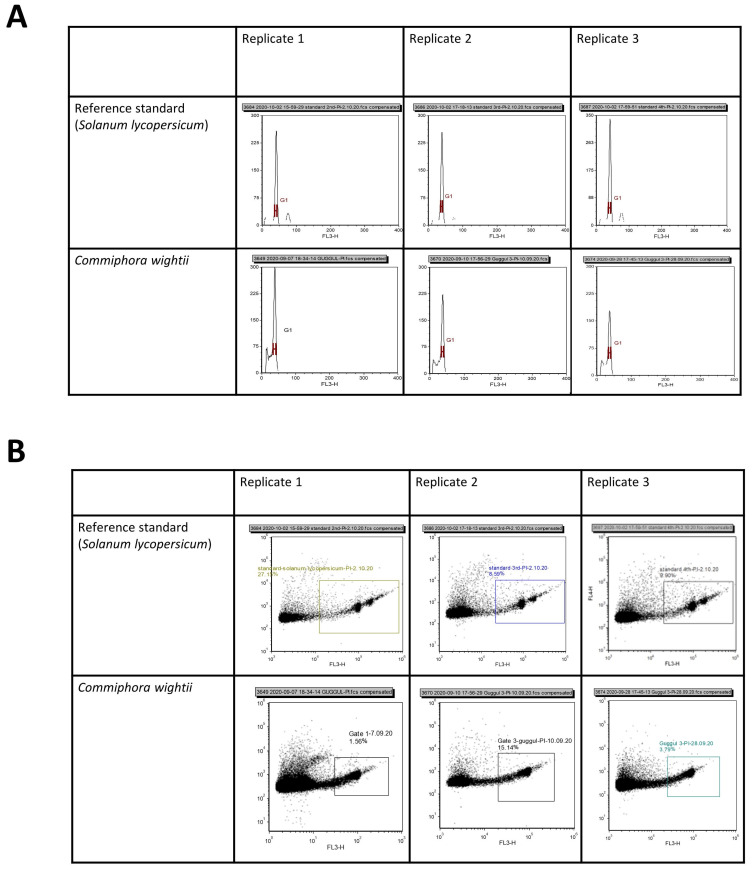
Flow cytometry analysis showing histogram (**A**) and density plots (**B**) of G_0_/G_1_ peaks of *Commiphora wightii* and reference standard *Solanum lycopersicum.*

**Figure 2:**
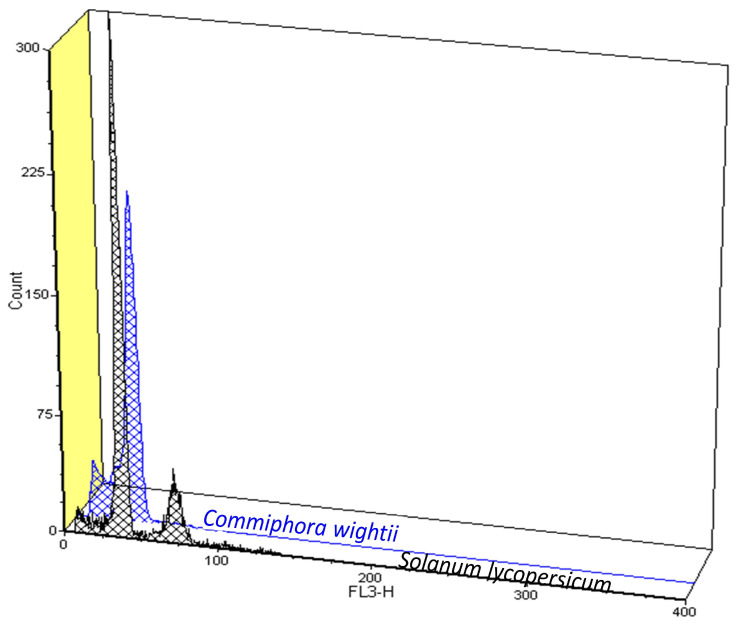
Flow cytometry data based on three-dimensional histogram plot showing the overlaying of G_0_/G_1_ peaks of *Commiphora wightii* and reference standard *Solanum lycopersicum.*

The histograms generated in the standardized FCM procedure resulted in G_0_/G_1_ peaks with coefficients of variation ranging from 4.20% to 5.45% (Figure 1A,B and Figure 2, Table 2). When compared with *Solanum lycopersicum* var. stupicke, as an internal reference standard, the 2C DNA and genome size of *C. wightii* was estimated at 1.85 pg and 904.63 Mb, respectively (Table 2), which showed negligible variation in size with the assembled draft genome (1.03 Gb).

### 3.3. De Novo Genome Assembly

Assembly of the genome resulted in 1.4 Gb with a contig N50 of 65,878 bp with CLC assembly (Supplementary Figure S3, Table 3). After assembling the sub-reads, post corrected draft genome size was 1.03 Gb with N50 of 74,387 bp (~74.4 Kb). Statistical analysis of base pairs in the corrected draft genome showed the average GC content in the draft genome was 35.6%.

### 3.4. Evaluation of Assembled Draft Genome

Comparison of gene set to the 138 genomes in the three lineage datasets (Viridiplantae, Eudicots and Embryophyta) resulted in 98.7% of total complete BUSCOs (C) (4307 complete BUSCO out of 4365) indicating that the genome assembly integrity was good. The total complete BUSCOs comprised 421 (99.1%) in Viridiplantae, 2293 (98.6%) in Eudicots and 1593 (98.7%) in Embryophyta lineages. The detailed single-copy BUSCOs (S), duplicated BUSCOs (D), fragmented BUSCO and missing BUSCO in three different plant lineage datasets are given in Table 4.

### 3.5. Repeat Analysis

The assembled draft genome had 342.35 Mb of transposable elements (repetitive DNA sequences against *Arabidopsis thaliana* dataset), constituting 33.1% of the total genome (Table 5). In the repeat sequences, the interspersed repeat constituted a major component of the genome (8.33%, ~86.18 Mb) besides ~1 Mb of tandem repeats. The sequenced genome had 85.9 Mb of transposon elements, in which ~83.9 Mb belonged to class I and ~1.92 Mb to class II elements. The total length of retro-elements was ~83.99 Mb covering 8.12% of total genome, while LTR elements constituted 8.12% with a total length of ~83.94 Mb. DNA transposons occupied 0.19% of the genome with a length of ~1.92 Mb. However, 1501 repeat elements with a total length of ~274 Kb remained unclassified, i.e., only 0.03% of the genome. In the tandem repeats, the total number of simple repeats was 9555, comprising 0.09% of genome with a length of ~0.91 Mb. In contrast, the number of small RNAs was 1504, occupying 0.07% of the genome (Supplementary Table S1).

### 3.6. Gene Prediction and Functional Annotation

A total of 31,187 genes were predicted and annotated from the assembled draft genome (Supplementary Table S2). The average length of the predicted genes and coding sequences (CDS) was 28694.46 bp and 6377.33 bp, respectively. The proteins had 2125.46 amino acids (Supplementary Tables S3 and S4). Comparing the predicted gene set of *C. wightii* with that of functional database revealed a total of 28,482 annotated genes that accounted for 91.32% of the total assembled draft genome. Annotation of the guggul draft genome with BLASTX showed the presence of sequence similarity with several other species. However, the top 10 organisms with a maximum sequence similarity were considered for organism annotation study. Comparative analysis suggested that *Vitis vinifera* has the maximum sequence similarity with number of hits reaching almost 6000 followed by *Citrus clementina* and *C. sinensis* with a value of 2501 and 2224, respectively (Supplementary Figure S4). *Morus notabilis* showed the least sequence similarity (579 hits).

**Figure 3:**
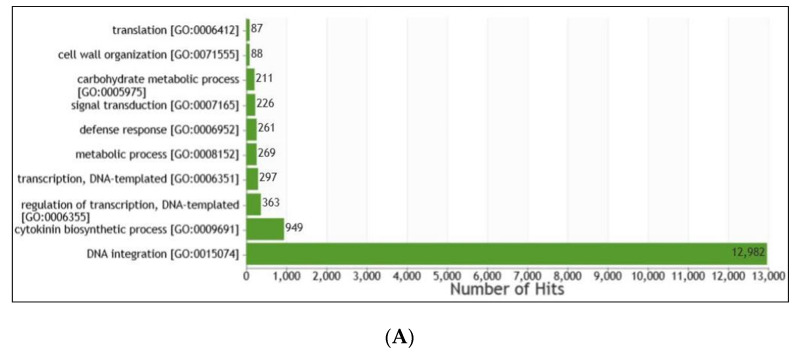

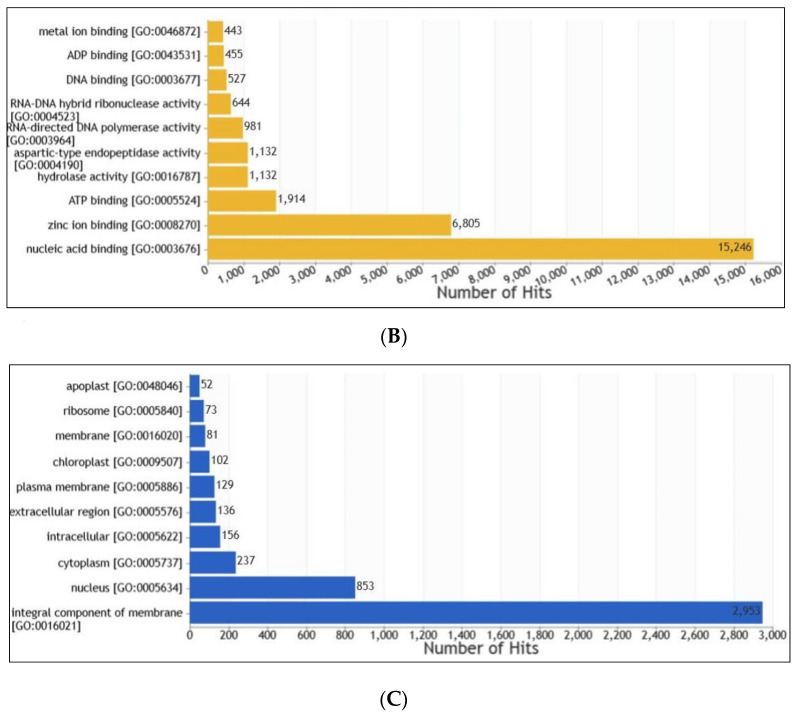
Gene ontology annotation of genes based on domains present in the encoded proteins. (**A**) Biological process classification, (**B**) molecular function classification and (**C**) cellular component.

The elucidation of gene ontology for biological processes, molecular function and cellular components was mapped using an in-house pipeline. Based on GO terms, identified genes were further mapped in three categories: biological process (BP), molecular function (MF) and cellular component (CC), respectively (Supplementary Table S5a–c). In the BP category regulation of transcription, DNA templated (GO:0006355) with 363 genes and a cytokinin biosynthetic process (GO:0009691) with 949 genes and DNA integration (GO:0015074) with a maximum of 12,982 genes were prominent. In the MF category, ATP binding (GO:0005524) with 1914 genes, zinc ion binding (GO:0008270) with 6805 genes and nucleic acid binding (GO:0003676) with 15,246 genes were highly represented. Cytoplasm (GO:0005737) with 237 genes, nucleus (GO:0005634) with 853 genes and integral component of membrane (GO:0016021) with a total of 2953 genes were dominant in the CC category (Figure 3).

### 3.7. Genes Associated with Primary and Secondary Metabolite Pathways

**Figure 4:**
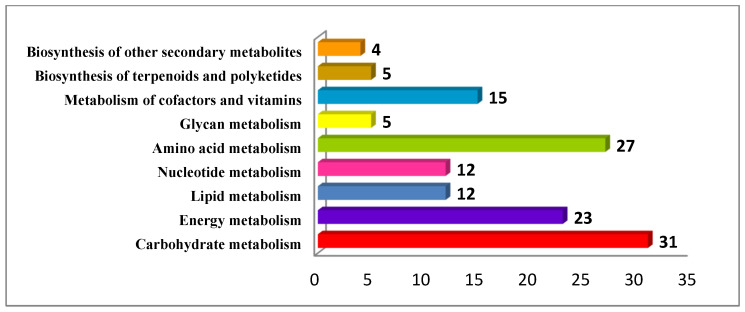
Major metabolic pathways identified in the genome of *C. weightii*. Number of genes associated with each pathway was shown adjacent to each bar.

The functional classification of the genes primarily involved in different metabolic pathways, KEGG (Kyoto Encyclopedia of Genes and Genomes), was performed using Blast2Go software (https://www.blast2go.com). A total of 1082 genes were mapped across 302 different metabolic pathways (Figure 4). Further, pathways were classified into 10 different pathway modules based on the KEGG’s canonical classes of pathway maps. These pathways were further grouped into two major categories: (1) metabolism of terpenoids and polyketides (two pathways, five genes) and (2) biosynthesis of other secondary metabolites (three pathways, four genes). The most enriched secondary metabolites characterized in the “Metabolism of terpenoids and polyketides” were terpenoid backbone biosynthesis and plant terpenoid biosynthesis. The most important pathways in the “Biosynthesis of other secondary metabolites” category were monolignol biosynthesis, flavanone biosynthesis and mugineic acid biosynthesis.

### 3.8. Abundance of Phytosterol Pathways Genes in C. weightii Draft Genome

Genes associated with the biosynthesis of phytosterol were analyzed. Cycloartenol synthase 1(CAS1) was found to be involved in phytosterol biosynthesis (Supplementary Figure S5). This key gene (seq id: g234.t1) had 83% matching with the *Arabidopsis* dataset (geneID sp|P38605|CAS1_ARATH). Thus, the key gene was unique with a 16% mismatch altogether. The other important gene (g116.t1) was matched with a 75% identity with the squalene epoxidase of *Arabidopsis* dataset [NC_003071.7: c9726384-9723615_SQE2_ARATH_GeneID=816814] involved in phytosterol biosynthesis.

The precursor of squalene epoxidase is squalene 2,3 epoxide, which diverted the pathway flux towards phytosterol biosynthesis through squalene epoxidase.

The HMGR [3-hydroxy-3-methylglutaryl-coenzyme A reductase 1, NC_015439.3:c46508678-46505935_HMGR_SOLLC_GeneID=543702] of *Solanum lycopersicum* was found to be mapped with 67.5% homology with *C. weightii* gene (g892.t1). There were also few known transcription factors (WRKY1, MYC2, involved on phytosterol biosynthesis genes were orthologous within the gene list of *Commiphora weightii* (Table 6).

### 3.9. Comparative Genomics and Phylogenetics

**Figure 5:**
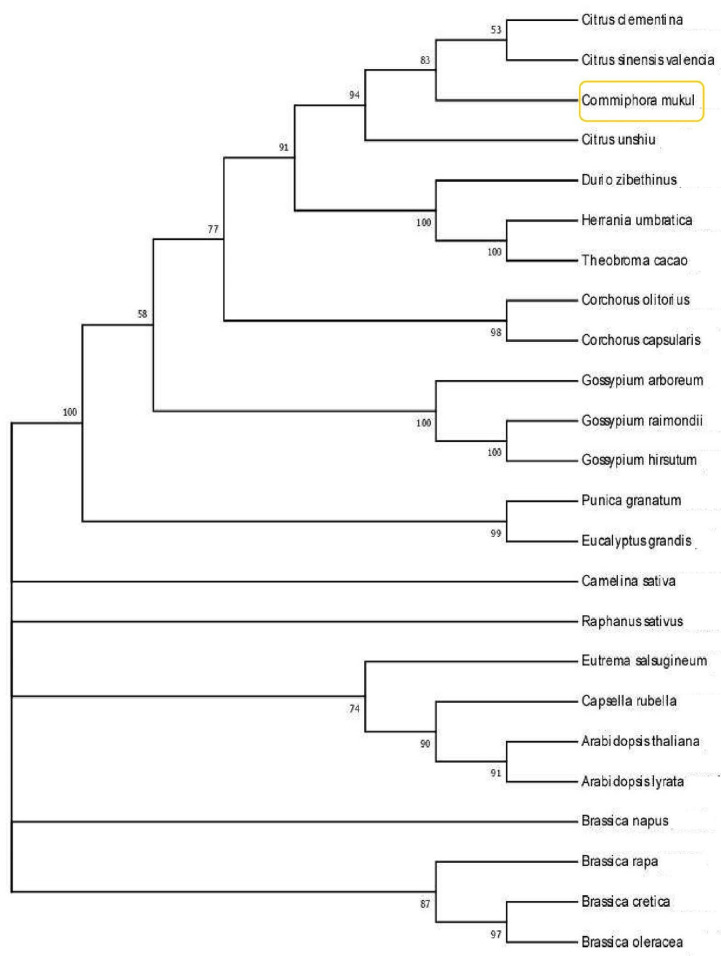
Phylogenetic relationship among 24 plant species generated from MEGA version 7.0 by using maximum parsimony algorithm with a bootstrap value of 1000.

To understand the evolutionary status of guggul, the molecular phylogeny was studied based on the whole genome at species level. The draft genome of *C. wightii* when compared with 23 available genomes in the public domain (Supplementary Table S6, Figure 5), revealed 2692 orthogroups. Species tree derived from the phylogenetic analysis indicated that *Citrus unshiu*, *C. sinesis* and *C. clementiana* in Rutaceae diversified earlier than *Commiphorawightii*, and *Citrus clementiana* and *C. sinesis* were closely related to *Commiphora wightii*. Phylogenetic analysis of *C. wightii* with other sapindales members such as *C. sinesis*, *C. clementiana* and *C. unshiu* revealed the common ancestry with members of Malvales such as *Corchorus capsularis*, *C. olitorius*, *Duriozibethinus*, *Gossypium arboreum*, *G. hirsutum*, *G. raimondii*, *Herraniaumbratica* and *Theobroma cacao*. In addition, information obtained from the phylogenetic analysis suggested that Sapindales and Brassicales are distantly related with each other and members of Brassicales diverged earlier than members of Malvales, Myrtales and Sapindales. Consequently, phylogeny revealed new information on origin and relatedness of *C. wightii* with its close relatives.

**Figure 6:**
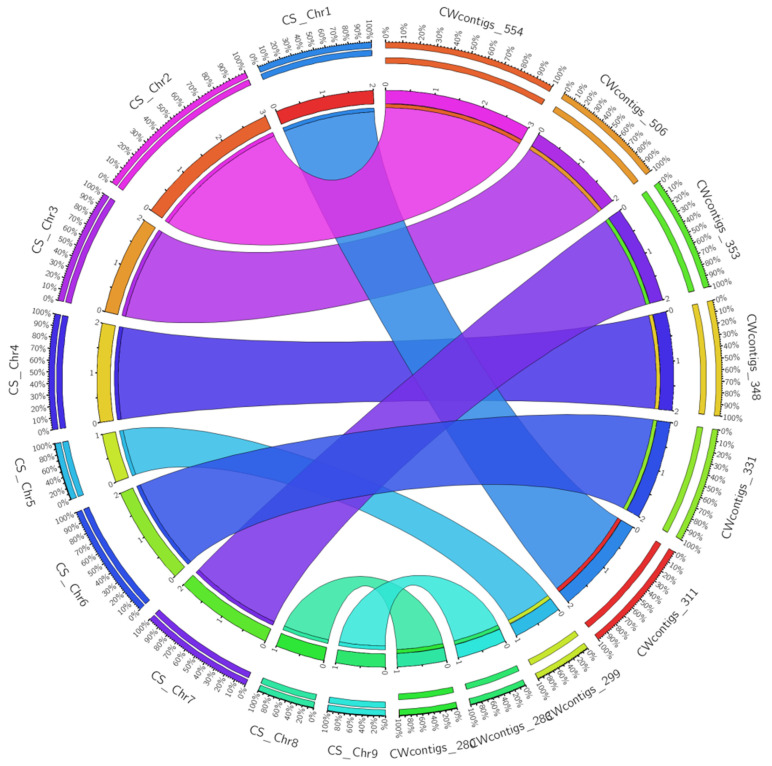
Circos plot obtained through synteny analysis by comparing assembled contigs of *Commiphora wightii* with *Citrus sinensis* reference genome; CS = *Citrus sinensis* and CW = *Commiphora weightii.* The number after contigs represents total number of syntenic blocks.

Synteny analysis using *Citrus sinensis* as a reference resulted in 3357 syntenic blocks altogether with more than 1kb hits. A total of 3265 synteny blocks of assembled contigs of *C. wightii* were collinear with the nine chromosomes and 92 with cp and mitochondria genome of *C. sinensis* (Supplementary Table S7). The assembled genome of *C. wightii* had a maximum number of synteny blocks (554) with the chromosome-2 of *C. sinensis*, followed by chromosome-3 (504) and the least number of synteny blocks were found in chromosome-8 and 9 of *C. sinensis* (Figure 6). Moreover, there were 3265 synteny blocks of the assembled draft genome of *C. weightii*, 24 (99.9% coverage) syntenic blocks in cpGenome and 68 (33.1% coverage) in mtGenome (Table 7). When all BLAST hits of assembled contigs of *C. weightii* with all chromosomes of *Citrus sinensis* were performed and mapped in the Circos plot, our results on micro-synteny blocks suggested that draft genome of *C. wightii* and publicly available reported genome of *C. sinensis* carry a strong genomic syntenic relationship (Supplementary Figure S6a–k).

### 3.10. Mining of SSR Markers

SSR markers were identified from the assembled draft genome. A total number of 23,822 sequences with ~1.03 GB were examined for SSR identification. Out of the 23,822 sequences, 22,294 were SSR-containing sequences with a total of 3,86,735 SSRs. Of these, 3,38,755 SSRs were in simple form and 47,980 were in compound form. With respect to number of repeat motifs, dinucleotide repeats (DNRs) were more abundant (80,992) than TNRs (24,606), TtNRs (3982) and PNRs (1724) (Supplementary Table S8).

## 4. Discussion

Whole genome sequencing of *Commiphora wightii* is crucial for elucidating pathways/genes associated with guggulsterone biosynthesis. Since no genome information is currently available in respect to *C. wightii*, the first-ever genome sequence reported in the present study, albeit being a draft genome, has high future relevance. The size of the *C. wightii* draft genome is approximately 1.03 GB. The presence of a large number of repetitive sequences in the genome, intricacies in the reproductive process, and the high level of heterozygosity made the assembling process challenging. Most of the plant species are scaffolded with genetic linkage maps into chromosome-scale assemblies. However, we could not achieve this in *C. weightii* because of constraints related to data availability for effective scaffolding, e.g., data on HiC, optimal map and genetic map [36,37,38]. Despite these constraints, our contig N50 was similar (N50:74.4 Kb) to that of the available draft genome assembly of other medicinal plants (e.g. N50:18.8 Kb *D. rotundata*, N50: 9.3 Kb in *E. guineensis* (N50:114.3 Kb in *A. comosus*) [36,37,38]. The assembled genome was also largely non-repetitive, indicating less redundancy in gene sequences. Yet the assembly generated in this study has ample scope for improvement to obtain a high quality genome of *C. weghtii* as characterized by high contig N50, high average read depth, and presence of most of the BUSCOs. The Hi-C sequence and optical map data would improve the *C. weightii* genome assembly to chromosome-scale. 

The estimated genome size of *C. wightii* was 1.03 Gb, making it one of the largest draft genome sequences that is currently available. Other species with large genomes include, *Cannabis sativa* (534 Mb), *Cajanus cajan* (833.07 Mb), *Azadirachta indica* (364 Mb), *Morus alba* (330 Mb), *Citrus sinensis* (367 Mb), *Quercus suber* (953.3 Mb) and *Asclepias syriaca* (420 Mb) [14,18,39,40,41,42,43]. Angiosperms are known to exhibit a significant amount of variation in their genome size that ranges from ~60 Mb in *Genlisea aurea* to ~150 Gb in *Paris japonica* [44,45]. Several factors contribute towards the variation in genome size resulting in genome purging or bloating. Among these, transposable elements are essential regulators that alter genome content [44]. The accumulation of transposable elements in the genome is caused due to epigenetic modifications such as DNA methylation. In recent times, several studies based on the genome-wide DNA methylation revealed the impact of epigenome in regulating genome size variation [44].

The “C-value paradox” as revealed from correlation analysis between FCM-based genome size estimation and genome assembly, suggested a disproportionate relationship between genome size and number of genes present on it. Though several studies have shown a positive correlation between the genome size and gene content in prokaryotes and primitive eukaryotes, such a trend is not consistent in higher eukaryotes [45,46,47]. Despite a three-fold increase in genome size (*Commiphora wightii* 1.03 Gb vs. *Citrus sinensis* 367 Mb), the number of predicted genes in *C. wightii* was 31, 187, which was quite similar to 29, 445 genes in *Citrus sinensis.* The total genome content (1C DNA) vs. the level of complexity in organisms still remains unexplained.

Sequencing the entire genome would include both repetitive and non-repetitive areas, increasing the genome size. Therefore, compared to the entire genome assembly, draft genome sequence always delivers a substantially smaller genome size. We were able to sequence only the regions that had a single copy of each sequence. The regions with repetitive sequences were excluded from the sequencing and assembly. The percentage of repeat elements in guggul was smaller than that in *Cajanus cajan* (51.67%) and *Morus alba* (47%), but greater than *Azadirachta indica* (13.03%) and *Citrus sinensis* (20.5%) genomes [14,18,40,41]. Variation in genome size among the closely related species not only provides essential clues to understand the underlying mechanisms of such variations but also helps to establish phylogenetic relationship within and among the species. However, presence of repetitive elements in the genome in high proportion does create complexities for analysis and annotation, limiting the characterization of some significant regions of the genome. These difficulties become more evident when conducting a comparative analysis of repeats among multiple species. Stress conditions and other environmental factors can up-regulate the activity of TEs and consequently introduce new insertions within the genome. As a result, the sequences at the flanking regions may become transcriptionally activated under similar type of stress conditions. Such type of regulatory mechanism exhibited by TEs may allow rapid genome evolution and enhance the ability of plants to withstand extreme environmental conditions. Further studies are required for elucidating the evolutionary dynamics of TEs and understanding their impact on plant genome size, organization, expression and evolution.

Our results revealed the presence of maximum number of genes related to DNA integration compared to other genes, suggesting a greater stability and integrity in the chromosomes. In addition, genes for nucleic acid binding and integral membrane component have also shown maximum numbers in terms of molecular functions. The study of Gene Ontology (GO) helped understanding the physiological adaptation of the species to survive in extreme environmental conditions.

Different contigs of the assembled draft genome of *C. wightii* showed a high level of synteny with most of the chromosomes of *Citrus sinensis,* suggesting structural similarity between *Citrus* and *Commiphora* genomes. In addition, synteny analysis also revealed high degree of stability in both the species, which might have conserved throughout the evolutionary process. Further, the presence ofhigh level of gene density and intergenic regions could have resulted in the structural similarities in both the genomes. The maximum number of syntenic blocks of *C. weightii* was collinear with the chromosome number 2 of *C. sinensis* followed by chromosomes 3. Conversely, the least number of orthologous regions were identified in chromosome 8 and 9 of *C. sinensis*. A comparison of syntenic blocks between the two species would enable us to study the structural and functional diversity that could lead to decipher complex evolutionary relationships.

The developed genomic resources such as SSRs could be used to get insights into the population structure of *C. wightii* using the identified SSRs to infer various levels of variation within and between populations. Further evaluation of these polymorphisms should help guggulsterone association studies. The simultaneous rearrangement of SSRs and the evolution of the plant genome can take place [48].

Several studies have been conducted in the field of plant sterol biosynthesis. However, there is little information on guggul biosynthesis. Plants mostly retain the two pathways, mevalonate synthesis and isopentenyl pyrophosphate (IPP) polymerization, although the post-squalene pathway shows noticeable modifications. A special oxidosqualenecyclase found only in plants, cycloartenol synthase (CAS), is able to catalyze the transformation of 2,3-oxidosqualene into cycloartenol, a pentacy clictriter penoid that serves as a common precursor for cholesterol and C24-alkyl sterols. By using this method, the cation may be moved from the oxidized squalene’s C-20 position to the position C-9, creating a cyclopropane ring between C-9 and C-19. Recent research has shown that CAS significantly affects sterol content and, as a result, changes phenotypic traits [49]. It is generally known that sterol side chain reductase (SSR) in plants is sub-divided into sterol side chain reductase 1 (SSR1) and sterol side chain reductase 2 (SSR2) [50]. According to earlier research, SSR1 catalyzed the final step in the synthesis of campesterol and sitosterol, but SSR2 is simply in charge of changing cycloartenol into cycloartenol in the cholesterol pathway [51,52]. The development of several types of plants’ reproductive and vegetative organs is reportedly affected by SSR1, also known as DFW1 [53].SSR2 contributes primarily to the production of cholesterol in plants, where it catalyzes the conversion of cycloartenol to cycloartenol, in contrast to SSR1′s function in the biosynthesis of C24-alkyl sterols [54]. The shift in cholesterol content caused by SSR2 regulation in the plant has been demonstrated to have an impact on the buildup of steroidal glycoalkaloids (SGAs). SSR1 and SSR2 are crucial sterol side chain reductases because they are needed for the production of C24-alkyl sterols and cholesterol, respectively.

Sterol methyltransferase (SMT) in plants catalyzes the side chain’s alkylation [53]. SMT directs post-squalene metabolic fluxes toward the production of cholesterol, campesterol, and sitosterol, which is crucial for the variety of plant sterols [55,56]. SMT1 (EC) and SMT2 (EC), which are both engaged in primary methylation and secondary methylation, are two different kinds of SMT found in plants [57]. SMT1 catalyzes the methylation of cycloartenol into 24-methylene cycloartenol as the initial step in adding methyl groups to the 24-desmethyl sterols. SMT2 helped to enhance the conversion of 24- ethylenelophenol into 24- ethylidenelophenol. The branch point of the synthesis of C24-methyl sterols and C24-ethyl sterols is 24-methylenelophenol, which is intriguing [57,58].

The synthesis of sterols in plants is reportedly regulated by the transcription factors (TF) WRKY TFs, MYC TFs and ERF TFs [59,60,61]. In our findings, a few important transcription factors viz., WRKY1, MYC2, and ERG5 involved in phytosterol biosynthesis genes were identified in the gene list of *Commiphora weightii*. WRKY1, a member of the WRKY TF family, controls the concentrations of secondary metabolite products in plants, including sterols and glycoalkaloids [62]. It is notable that WRKY1, MYC2 and ERF4 are all involved in regulating sterol synthesis in plants whereas WRKY1 is a salicylic acid response factor, and MYC2 and ERF4 are all jasmonate acid response factors. Therefore, it is necessary to look into other transcription factors that are involved in the creation of plant sterols.

The main aspect of investigation into the post-transcriptional regulation of sterols is hydroxymethyl glutaryl-CoA reductase (HMGR), a rate-limiting enzyme in the plant sterol production pathway [63]. Therefore, we propose that plants regulate sterol variations in response to environmental stress, specifically changes in C24-alkyl sterol concentration, which may be implicated in hormone induction.

The over-extraction and harvesting of oleogum resin in an unsustainable manner have caused the depletion of *C. wightii* populations in nature. Further, other anthropogenic disturbances such as altered land use practices have caused habitat loss and fragmentation within the existing populations of *C. wightii* [64]. In critically endangered species with severely reduced populations, conventional breeding programmes may not be successful. The genome data is essential for precision breeding that would help the species to avoid such inbreeding related challenges as elimination of recessive lethal alleles and decreased disease resistance [65]. Previous studies have successfully developed in vitro propagation protocol for the species with anther culture and somatic embryogenesis [66]. Vegetative propagation through stem cuttings was also effective [64,67]. However, survival rate of the plants under field conditions remained poor and thus considered to be one of the major limitations for conserving the species. Only a limited number of studies focusing on the conservation through molecular/genetics approach are available [68,69]. Reduction in the effective population size and increase in homozygosity could be the two major driving forces for loss of diversity within and across the populations of *C. wightii*. For any conservation program, a dense and large number of markers are needed as the genomic resources. Information gained from genome sequencing programs can contribute towards conservation, and management of guggul. Genome sequencing can also help in understanding the evolutionary trends and prioritization of the imperiled populations for conservation.

## 5. Conclusions and Future Directions

The draft genome of *C. wightii* was put together using a hybrid assembly method, which revealed a size of ~1.03 Gb. The present study is the first attempt to sequence the draft genome of any member of the Burseraceae family. The variation in the size of *C. wightii*’s genome compared to that of other closely related species raises the possibility that the genome is influenced by a significant number of repetitive sequences. *Citrus* clade was revealed as a common ancestor for *C. wightii*, and the existence of more syntenic blocks between these two species further demonstrated the gene structural homology. In order to create superior varieties through focused molecular breeding programmes, understanding structural genomics is crucial.

The draft genome will be a valuable resource for further genomic studies in guggul. The draft genome would provide insight into the ecological adaptation of the species under the changing environmental conditions. In addition, the genomic resource will be helpful in designing an effective conservation programme including genomic-based conservation.

Future research should exploit the potential of whole genome sequencing and optical mapping to pinpoint the sequences of the genome’s gap regions. Refined complete genome could aid in identifying key genes, transcription factors, repeats, etc. with crucial roles in the biosynthesis of guggul/guggulsterone and species’ adaptability. The comparative metabolomics of guggul and its close relatives may reflect valuable insights into the convergence of secondary metabolites in order to identify the similarities in terms of chemical compounds. This approach might enable us to draw a conclusion on the plesiomorphic or apomorphic origin of this diverse class of secondary metabolites, which could be helpful in the elucidation of secondary metabolites in *C. wightii*.

Due to a lack of transcriptomics data, the structural variations within the genome with their functional consequences could not be correlated in the present study. Future studies should also focus on the expression of pathway gene/s associated with guggulsterone biosynthesis across different populations. Additionally, a transcriptome profile would be beneficial for locating defense responsive genes that could have a major impact on reducing biotic stress in the species.

## Figures and Tables

**Table 1:** Details of sequencing data, N50 value and quality score of *Commiphora wightii* using PacBio and Illumina sequencing platforms.

**Details of Long Read Sequencing** **(PacBio Platform)**	**1 SMRT**	**2 SMRT**		**3 SMRT**
Polymerase read bases (GB)	11.10	11.28		10.60
Polymerase reads	868,985	811,704		844,834
Polymerase read length (mean)	12,781	13,904		12,556
Polymerase read N50	23,750	24,250		22,750
Mean insert length (in bp)	9249	10,200		9446
Insert N50	15,750	16,250		15,750
**Details of short read sequencing** **(Illumina platform)**	**Read Orientation-1**		**Read Orientation-2**	
Mean read quality (Phred score)	39.35		38.02	
Number of reads	413,727,546		413,727,546	
% GC	43.57		43.65	
% Q < 10	0.15		0.49	
% Q 10–20	1.61		3.78	
% Q 20–30	2.62		4.9	
% Q > 30	95.62		90.83	
Number of bases (GB)	62.05913		62.05913	
Mean read length (bp)	150.0		150.0	

**Table 2:** Details of genome size estimation of *Commiphora wightii* with reference standard *Solanum lycopersicum* using flow cytometry.

	Replicate 1		Replicate 2		Replicate 3		Mean	2C DNA Content (pg)	Genome Size (Mb)
	Peak Median	CV%	Peak Median	CV%	Peak Median	CV%			
Reference standard (*Solanum lycopersicum*)	103,443	3.88	99,061	3.93	104,796	4.10	102,433.3	1.96	958.4
*Commiphora wightii*	98,194.50	5.45	96,821	4.96	95,020	4.20	96,678.5	1.85	904.65

**Table 3:** Details of the assembled draft genome of *Commiphora wightii* showing the total number of contigs, their size range and total length.

Size Range (bp)	Number of Contigs	Total Length (bp)
≥0	23,822	1,034,226,503
≥1000	23,822	1,034,226,503
≥5000	22,290	1,028,437,576
≥10,000	19,403	1,006,888,612
≥25,000	11,910	878,025,564
≥50,000	5,974	668,139,253

**Table 4:** Details of the orthologous sequences obtained through BUSCO analysis (Dependencies and versions: hmmsearch: 3.1, bbtools: 38.98, metaeuk: 6.a5d39d9, BUSCO: 5.4.2).

Parameters	Total Length (bp)	Percentage (%)	Lineage Dataset
Complete BUSCOs (C)	421	99.1	Viridiplantae_odb10 (Creation date: 10 September 2020, Number of Genomes: 57, Number of BUSCOs: 425)
Complete and single-copy BUSCOs (S)	135	31.8	
Complete and duplicated BUSCOs (D)	286	67.3	
Fragmented BUSCOs (F)	1	0.2	
Missing BUSCOs (M)	3	0.7	
Total BUSCO groups searched	425	100	
Complete BUSCOs (C)	2293	98.6	Eudicots_odb10 (Creation date: 10 September 2020, Number of genomes: 31, Number of BUSCOs: 2326)
Complete and single-copy BUSCOs (S)	686	29.5	
Complete and duplicated BUSCOs (D)	1607	69.1	
Fragmented BUSCOs (F)	14	0.6	
Missing BUSCOs (M)	19	0.8	
Total BUSCO groups searched	2326	100	
Complete BUSCOs (C)	1593	98.7	Embryophyta_odb10 (Creation date: 10 September 2020, Number of genomes: 50, Number of BUSCOs: 1614)
Complete and single-copy BUSCOs (S)	463	28.7	
Complete and duplicated BUSCOs (D)	1130	70.0	
Fragmented BUSCOs (F)	13	0.8	
Missing BUSCOs (M)	8	0.5	
Total BUSCO groups searched	1614	100	

**Table 5:** Summary of the assembled draft genome of *Commiphora wightii.*

Parameters	Measure
Estimated genome size (Gb)	1.03
Chromosome number (2n)	26
Total size of assembled contigs (Gb)	32.98
Number of contigs	107,221
Largest contigs (bp)	1,627,014
No. of predicted genes	31,187
N50 length of contigs (bp)	74,387
N75 length of contigs (bp)	36,142
GC content (%)	35.6
Total size of transposable elements (Mb)	342.35 (33.1%)

**Table 6:** Details of homology search for public domain phytosterol biosynthesis-associated genes in the predicted genes of *Commiphora weightii.*

Query ID	Subject ID	Percentage of Identical Matches	Alignment Length	Number of Mismatches	Number of Gap Openings	Start of Alignment in Query	End of Alignment in Query	Start of Alignment in Subject	End of Alignment in Subject	Expected Value
sp|P38605|CAS1_ARATH	g234.t1	78.357	767	127	2	1	758	245	981	0
NC_003071.7:c9726384-9723615_SQE2_ARATH_ [GeneID=816814]	g116.t1	75	340	60	1	1243	2262	3231	3545	2.42 × 10^−155^
NC_015439.3:c46508678-46505935_HMGR_SOLLC_ [ GeneID=543702]	g892.t1	67.5	314	67	4	124	1065	402	680	8.07 × 10^−120^
NW_023590956.1:3889228-3894037_SQE_JATCU_ [ GeneID=105630804]	g116.t1	65.2	247	25	1	3397	4137	3360	3545	5.67 × 10^−84^
NC_015449.3:c67067036-67062030_CPI_SOLLC_ [ GeneID=100301930]	g451.t1	64.4	45	16	0	4733	4599	654	698	6.81 × 10^−8^
NC_003075.7:17743738-17746697_SQE3_ARATH_ [ GeneID=829932]	g116.t1	60.9	455	115	7	1182	2546	3173	3564	2.24 × 10^−153^
NC_015438.3:2174569-2178590_CYP51_SOLLC_ [ GeneID=100736446]	g546.t1	56.8	738	63	2	1666	3876	2482	2964	1.01 × 10^−240^
NC_003075.7:16538189-16542024_SQS1_ARATH_ [ GeneID=829616]	g331.t1	52.9	206	25	3	1805	2422	158	291	2.12 × 10^−49^
NC_012015.3:c22173595-22152025_LOC100254746_VITVI_ [ GeneID=100254746]	g1271.t1	52.4	1484	443	24	1964	6328	11	1260	0
NC_003070.9:28695760-28698852_HMG1_ARATH_ [ GeneID=843982]	g892.t1	51.7	694	179	12	327	2408	404	941	1.70 × 10^−184^
NC_015438.3:3419728-3429015_LOC101248602__SOLLC_ [ GeneID=101248602]	g1497.t1	48.2	85	33	3	3083	3313	1994	2075	7.14 × 10^−09^
NC_003076.8:9690428-9693289_MK_ARATH_ [GeneID=832804]	g117.t1	46.3	374	138	11	1243	2355	5686	5999	4.25 × 10^−69^
NC_003070.9:c29688800-29684236_BAS_ARATH_ [GeneID=844234]	g314.t1	38.2	927	192	25	645	3422	10,225	10,771	3.15 × 10^−156^
NC_003070.9:20763844-20765823_LAS_ARATH_ [GeneID=842007]	g1051.t1	38	382	197	8	383	1528	15,772	16,113	3.60 × 10^−57^
NC_003074.8:c16517781-16512170_LAS1_ARATH_ [ GeneID=823649]	g234.t1	34.1	920	282	22	1972	4716	373	973	4.25 × 10^−119^
NC_003075.7:16542242-16545194_SQS2_ARATH_ [ GeneID=829617]	g331.t1	29	518	119	13	584	2137	78	346	1.30 × 10^−39^
sp|Q39227|SMT2_ARATH	g234.t1	87.24	337	43	0	1	337	4163	4499	0
sp|A0A0A1C3I2|HMGR1_PANGI	g892.t1	80.47	553	86	5	32	567	394	941	0
sp|A0A3Q7HRZ6|MYC2_SOLLC	g713.t1	60.197	711	207	19	1	686	3747	4406	0
sp|Q9SI37|WRKY1_ARATH	g355.t1	44.484	281	128	8	101	374	3523	3782	2.42 × 10^−60^
sp|Q39085|DIM_ARATH	g585.t1	38.043	92	55	2	118	208	56,779	56,869	0.0000861
sp|P45434|SSRA_ARATH	g343.t1	33.333	69	36	2	34	94	238	304	0.48
sp|G1UB11|ERG5_CANAL	g337.t1	27.273	517	345	13	10	511	942	1442	4.77 × 10^−50^

**Table 7:** Details of synteny block analysis of assembled contigs of Commiphora weightii with reference genome, Citrus sinensis [Citrus sinensis cultivar Valencia sweet orange, DVS_A1.0; Chr1=NC_068556.1; Chr2=NC_068557.1; Chr3=NC_068558.1, Chr4=NC_068559.1; Chr5=NC_068560.1; Chr6=NC_068561.1; Chr7=NC_068562.1; Chr8=NC_068563.1 and Chr98=NC_068564.1}. The cut-off value of query coverage of assembled contigs was synteny block in the analysis.

Reference Genome (*C. sinensis*)	Total Size (bp)	Total Mapped Query (bp)	Number of Syntenic Blocks	% of Syntenic Coverage
cpGenome	160,129	160,036	24	99.9
mtGenome	640,906	199,272	68	31.1
Chr1	24,849,987	501,459	311	2.0
Chr2	32,942,340	882,051	554	2.7
Chr3	52,308,633	830,996	506	1.6
Chr4	29,627,743	552,554	348	1.9
Chr5	38,998,145	494,795	299	1.3
Chr6	26,176,707	554,325	331	2.1
Chr7	29,493,366	597,503	353	2.0
Chr8	30,582,302	436,388	280	1.4
Chr9	33,998,288	483,919	283	1.4

## Data Availability

NCBI repository; SRA accession number for Ilumina: SRR12931174 and PacBio: SRR12931173, SRR12931172 and SRR12931171; Bio-project accession number: PRJNA645081; BioSample accession number: SAMN15491827). The data can be accessed by following the link: https://dataview.ncbi.nlm.nih.gov/object/PRJNA645081?reviewer=kmjuh8jqst2446f2bc990r6d9k.

## Supplementary Materials

The following supporting information can be downloaded at: https://www.mdpi.com/article/10.3390/life13030662/s1, Supplementary Figure S1: Polymerase read (A–C) and estimated insert lengths (D–F) obtained through long read sequencing in PacBio platform by using three SMRT flow cells namely 1SMRT, 2SMRT and 3SMRT respectively. Supplementary Figure S2: Graphical representation of short read sequencing, depicting the quality, base and GC distribution in read orientation 1 (A–C) and read orientation 2 (D–F), respectively. Supplementary Figure S3: Graphical representation showing total number of contigs and their respective size ranges in base pair unit. Supplementary Figure S4: Distribution of top ten organisms for organism annotation obtained through BLASTX program. Supplementary Figure S5: Genes involved in phytosterol biosynthesis pathways. Supplementary Figure S6a–j: Details of microsynteny blocks of each assembled contigs of *C. weightii* with all chromosomes (Chr-1-9 and mt-genome and cp-genome) of *C. sinenesis*. Supplementary Table S1: Details of the repeat analysis performed through RepeatMasker v 2.2 using *Arabidopsis* as reference. Supplementary Table S2: List of predicted genes with annotation. Supplementary Table S3: FASTA formatted genes with CDS region. Supplementary Table S4: FASTA formatted protein coding regions. Supplementary Table S5a–c: Details of GO annotation of predicted genes. Supplementary Table S6: Total number of 23 plant species selected as reference for constructing the phylogenetic tree. Each branch indicates the confidence level interval for each divergence. Supplementary Table S7: Details Synteny analysis of contigs of *C. weghtii* with homology search with *Citrus sinensis*. Supplementary Table S8: Results showing the total number of SSRs found in the draft genome of *Commiphora wightii* along with their distribution into different classes.
