# Supplementary material for: De Novo Hybrid Assembled Draft Genome of Commiphora wightii (Arnott) Bhandari Reveals Key Enzymes Involved in Phytosterol Biosynthesis
Source: Life (Basel). 2023 Feb 28;13(3):662. doi: 10.3390/life13030662 (PMC10052710; doi:10.3390/life13030662)
Supplement: Supplementary file 1 [file life-13-00662-s001.zip › life-2022038-supplemental Figures_MDPI Life_Guggul.pdf]

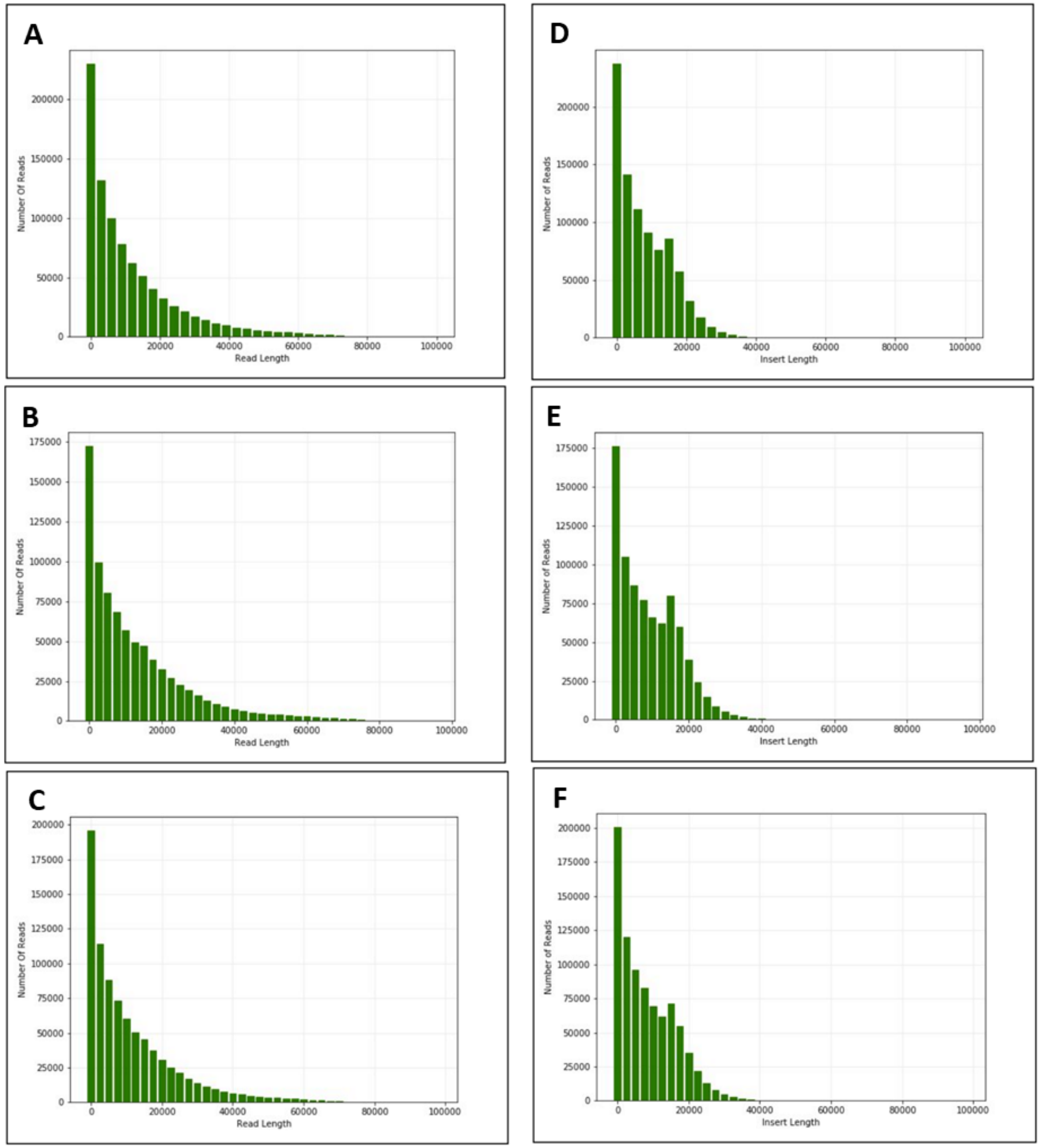

Supplemental Figure S1. Polymerase read (A-C) and estimated insert lengths (D-F) obtained through long read sequencing in PacBio platform by using three SMRT flow cells namely 1SMRT, 2SMRT and 3SMRT respectively

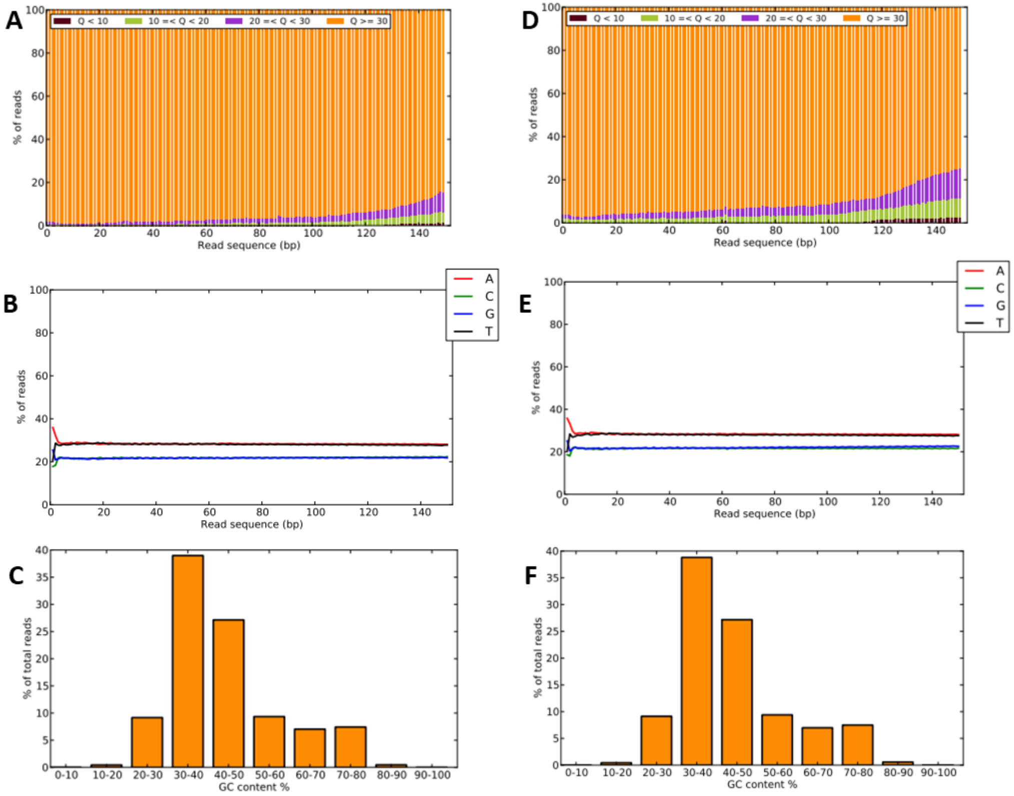

Supplemental Figure S2 Graphical representation of short read sequencing,depicting the quality, base and GC distribution in read orientation 1 (A-C) and read orientation 2 (D-F), respectively

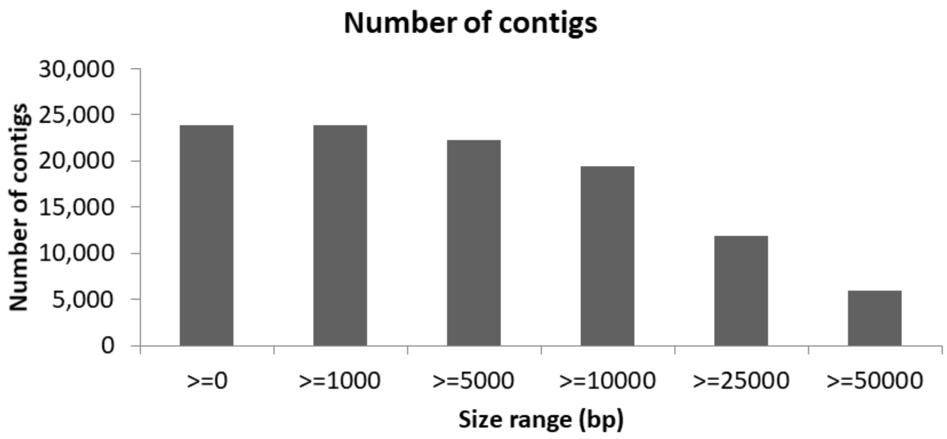

Supplemental Figure S3. Graphical representation showing total number of contigs and their respective size ranges in base pair unit

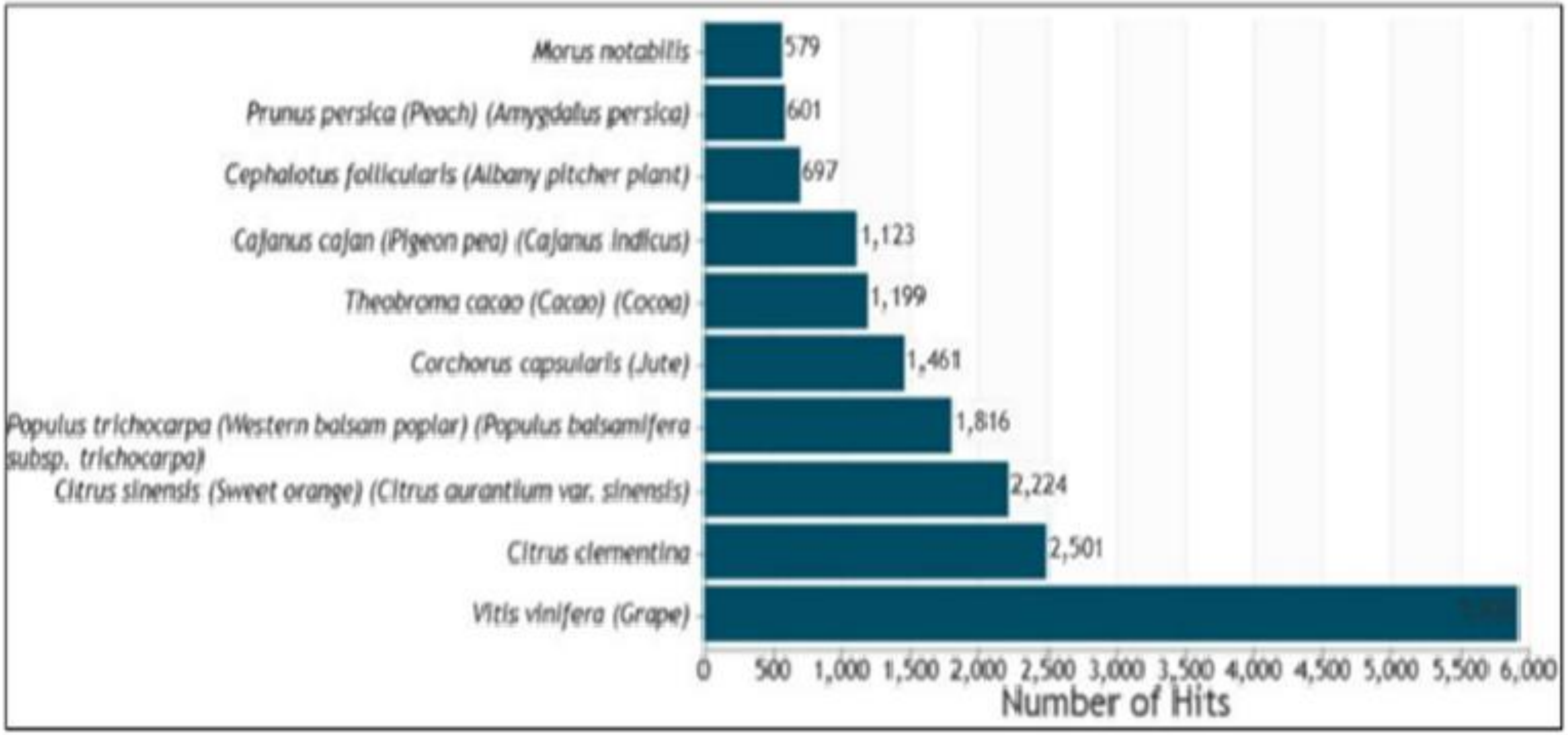

Supplemental Figure S4 Distribution of top ten organisms for organism annotation obtained through BLASTX program.

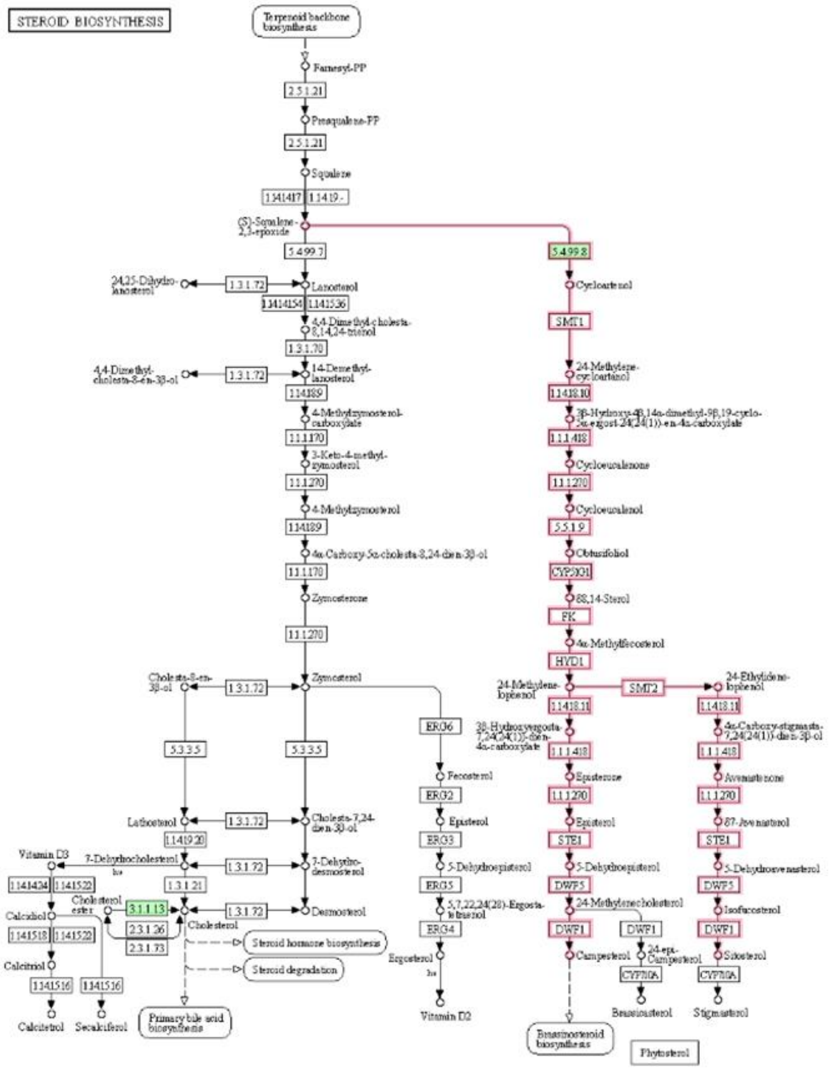

Supplemental Figure S5 Genes involved in phytosterol biosynthesis pathways

Chr-1

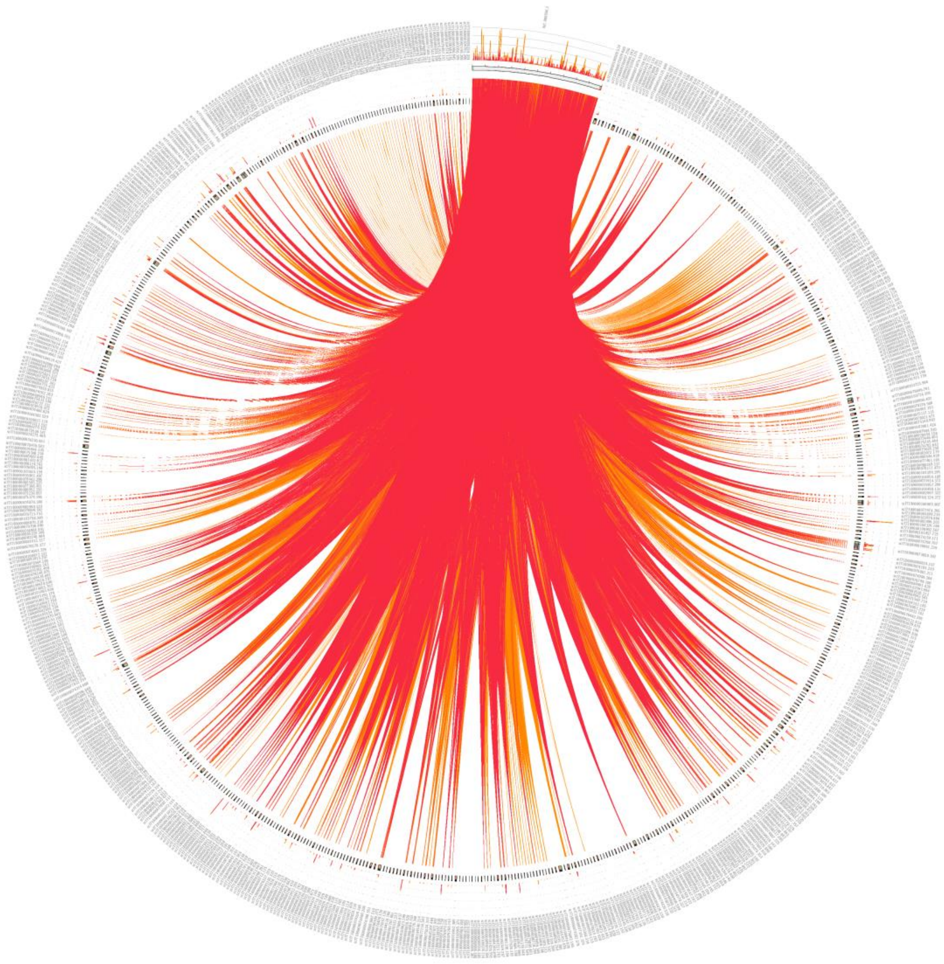

Supplemental Figure S6a Details of microsynteny blocks of assembled contigs of *C. weightii* with chromosome-1 of *C. sinenesis*

Chr-2

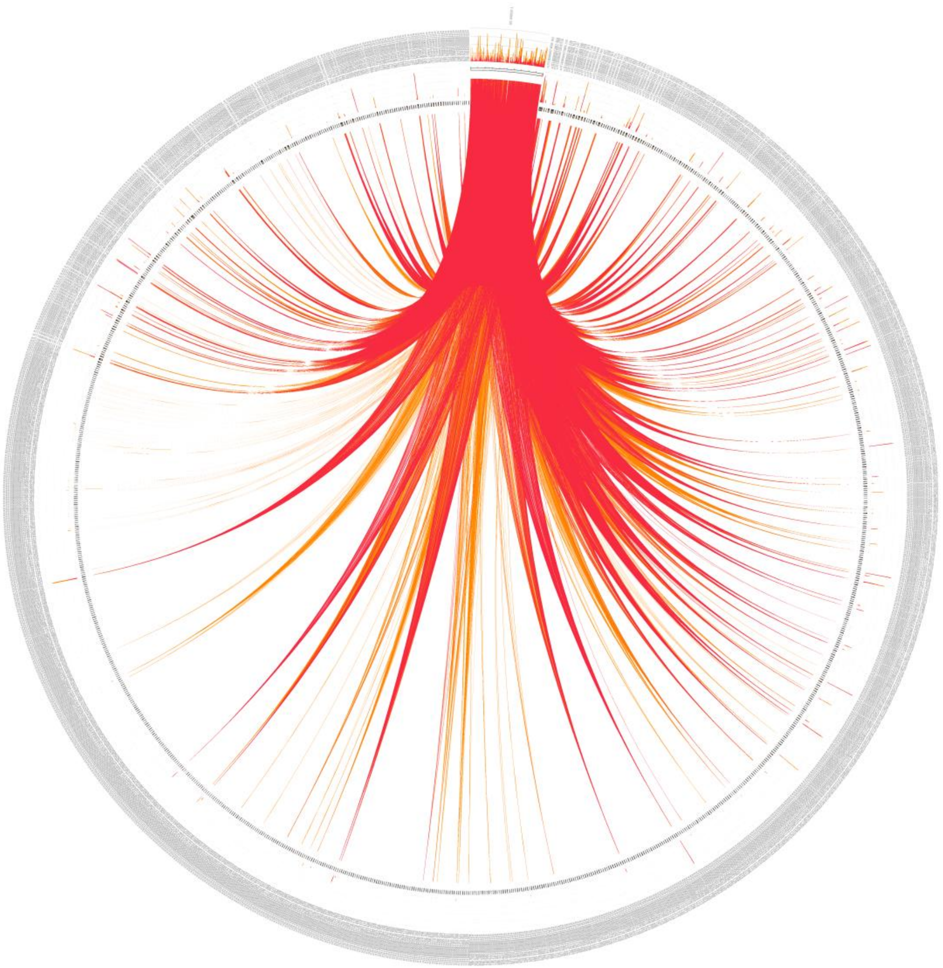

Supplemental Figure S6b Details of microsynteny blocks of assembled contigs of *C. weightii* with chromosome-2 of *C. sinenesis*

Chr-3

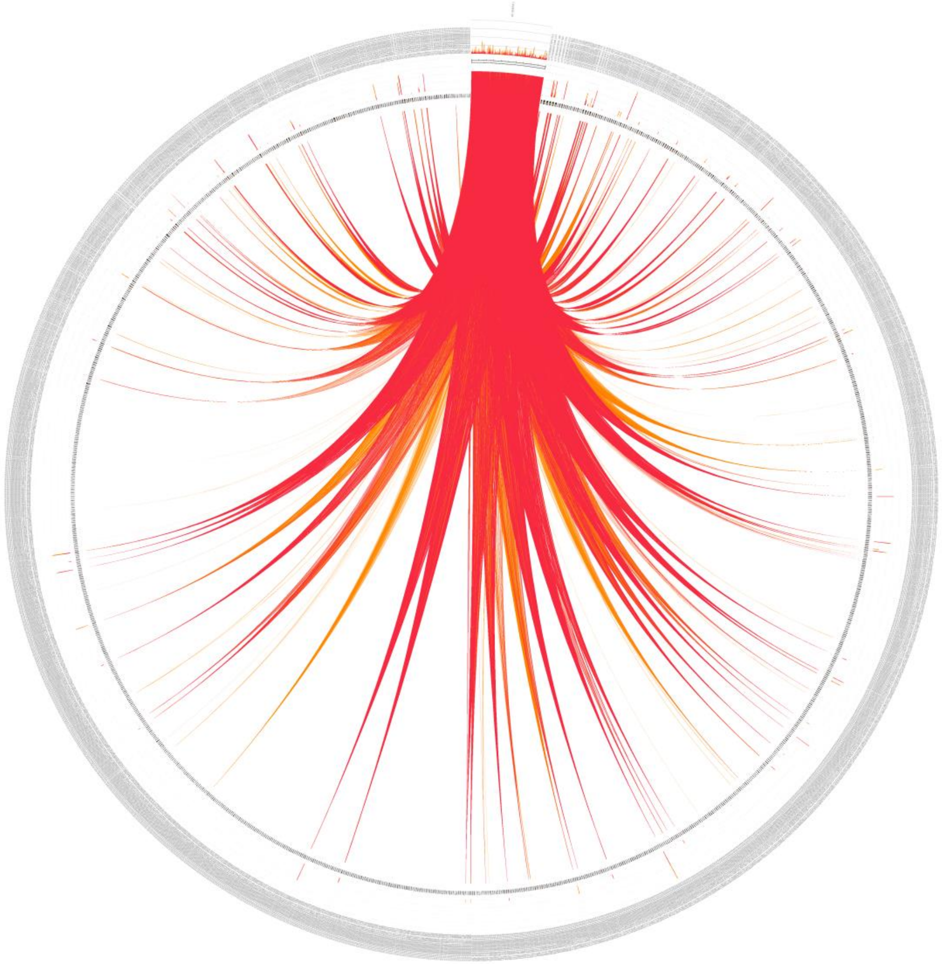

Supplemental Figure S6c Details of microsynteny blocks of assembled contigs of *C. weightii* with chromosome-3 of *C. sinenesis*

Chr-4

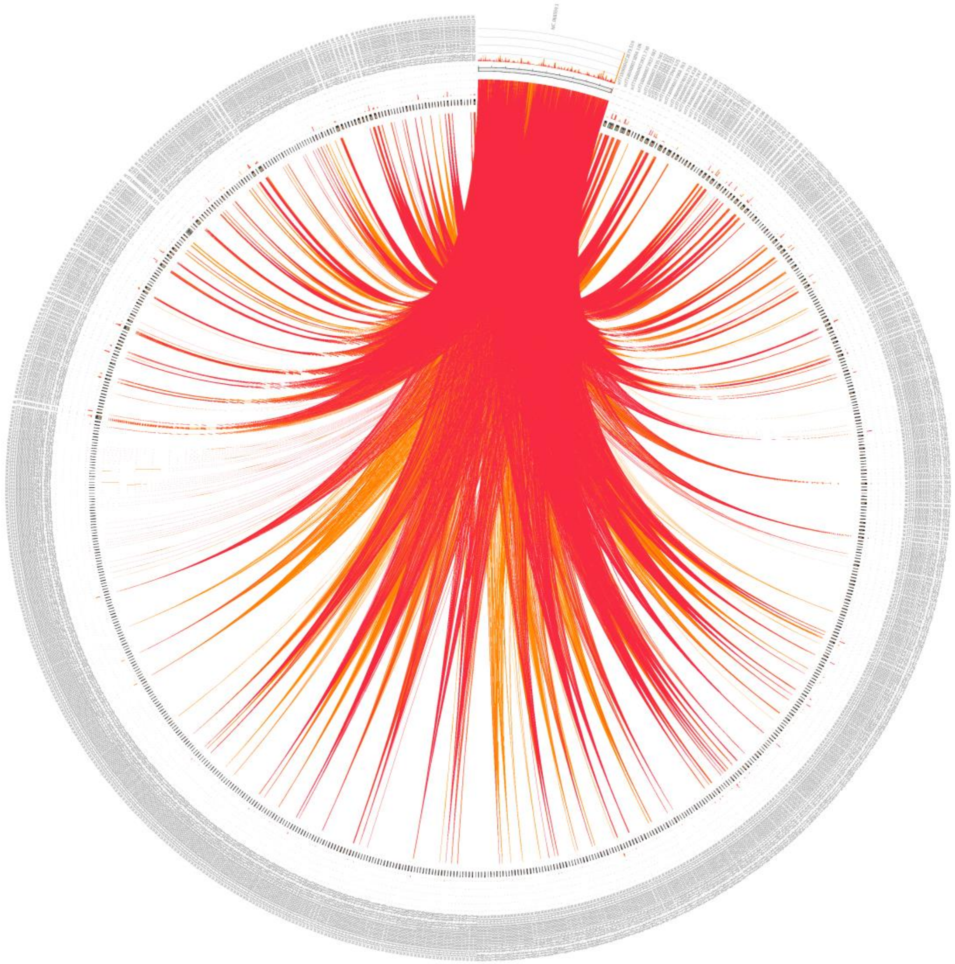

Supplemental Figure S6d Details of microsynteny blocks of assembled contigs of *C. weightii* with chromosome-4 of *C. sinenesis*

Chr-5

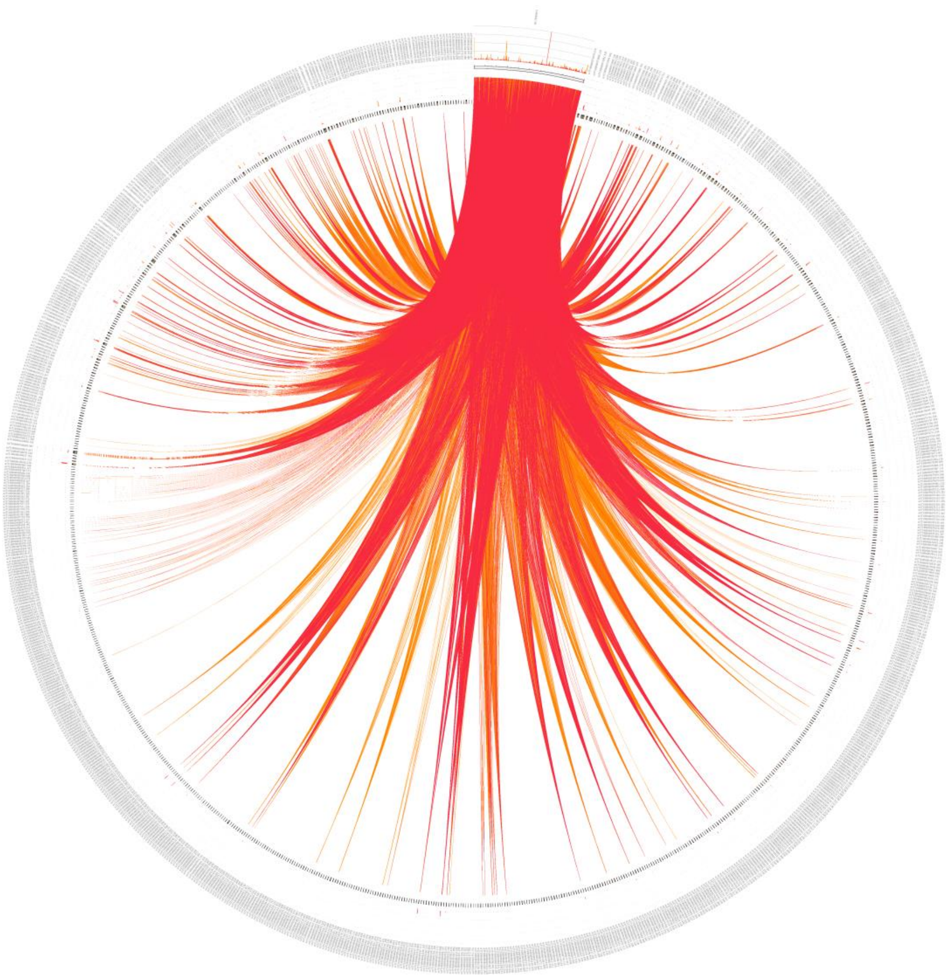

Supplemental Figure S6e Details of microsynteny blocks of assembled contigs of *C. weightii* with chromosome-5 of *C. sinenesis*

Chr-6

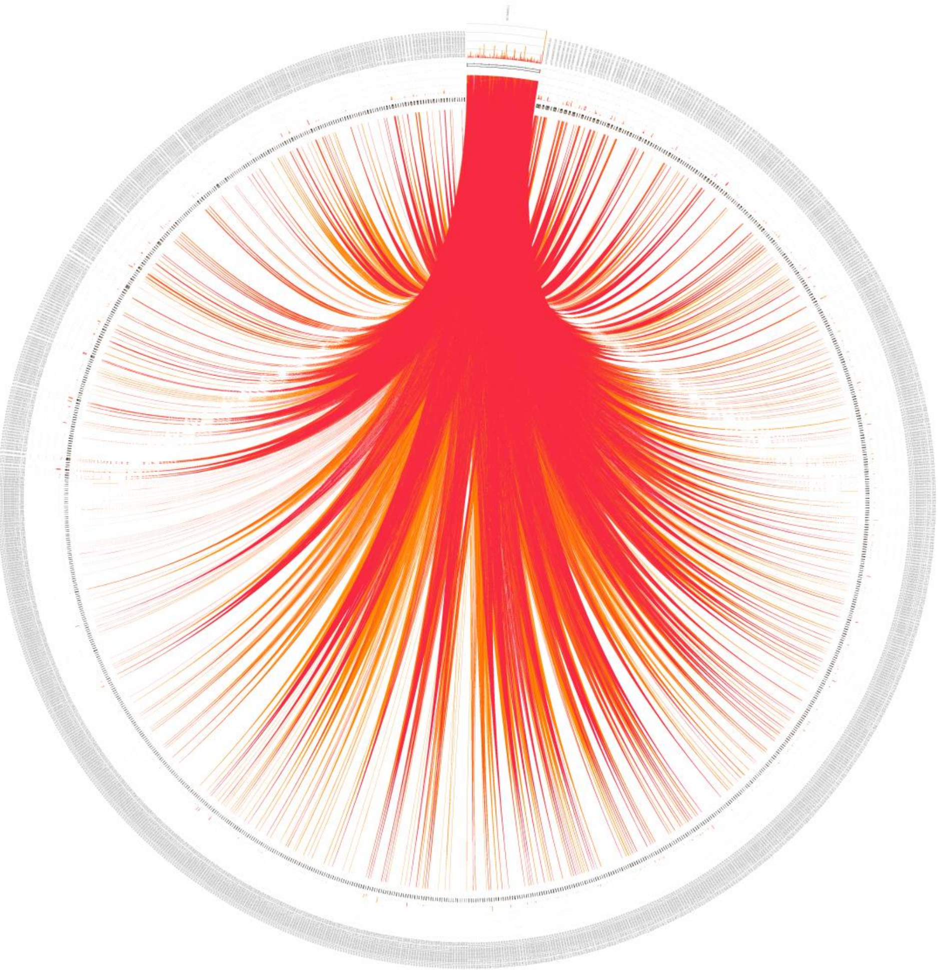

Supplemental Figure S6f Details of microsynteny blocks of assembled contigs of *C. weightii* with chromosome-6 of *C. sinenesis*

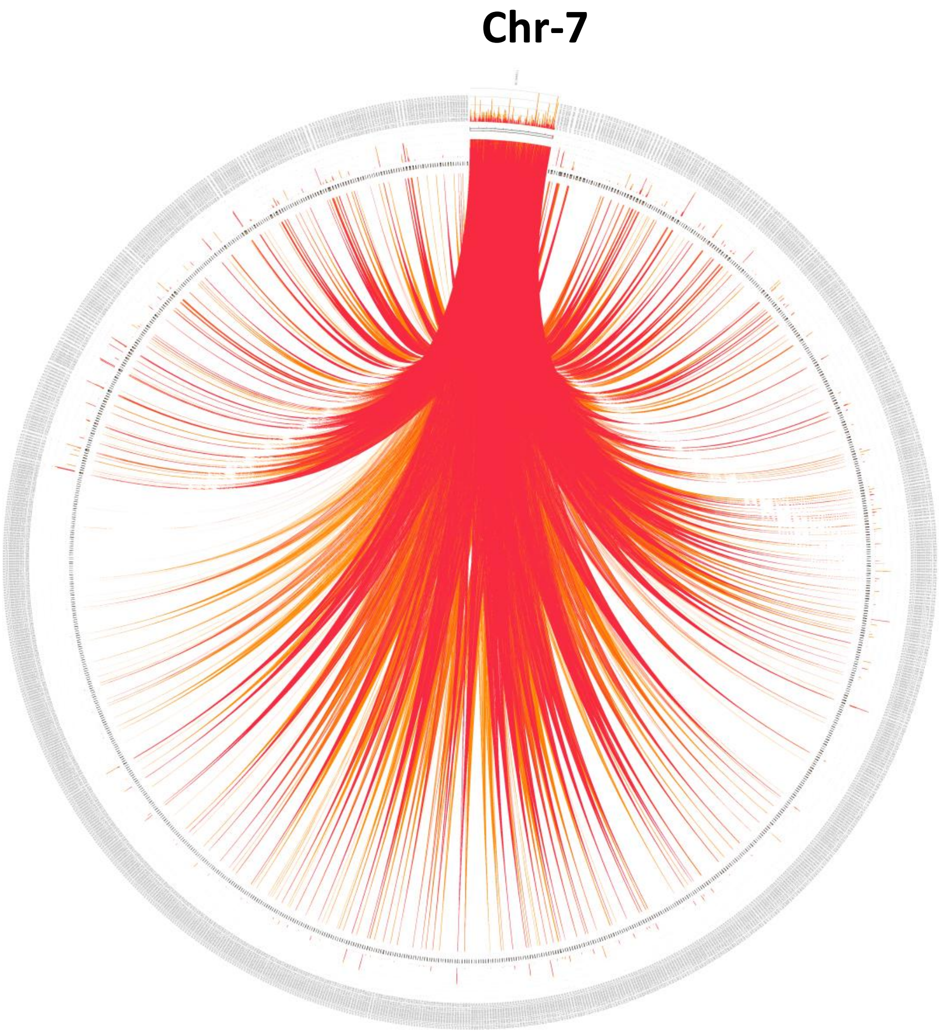

Supplemental Figure S6g Details of microsynteny blocks of assembled contigs of *C. weightii* with chromosome-7 of *C. sinenesis*

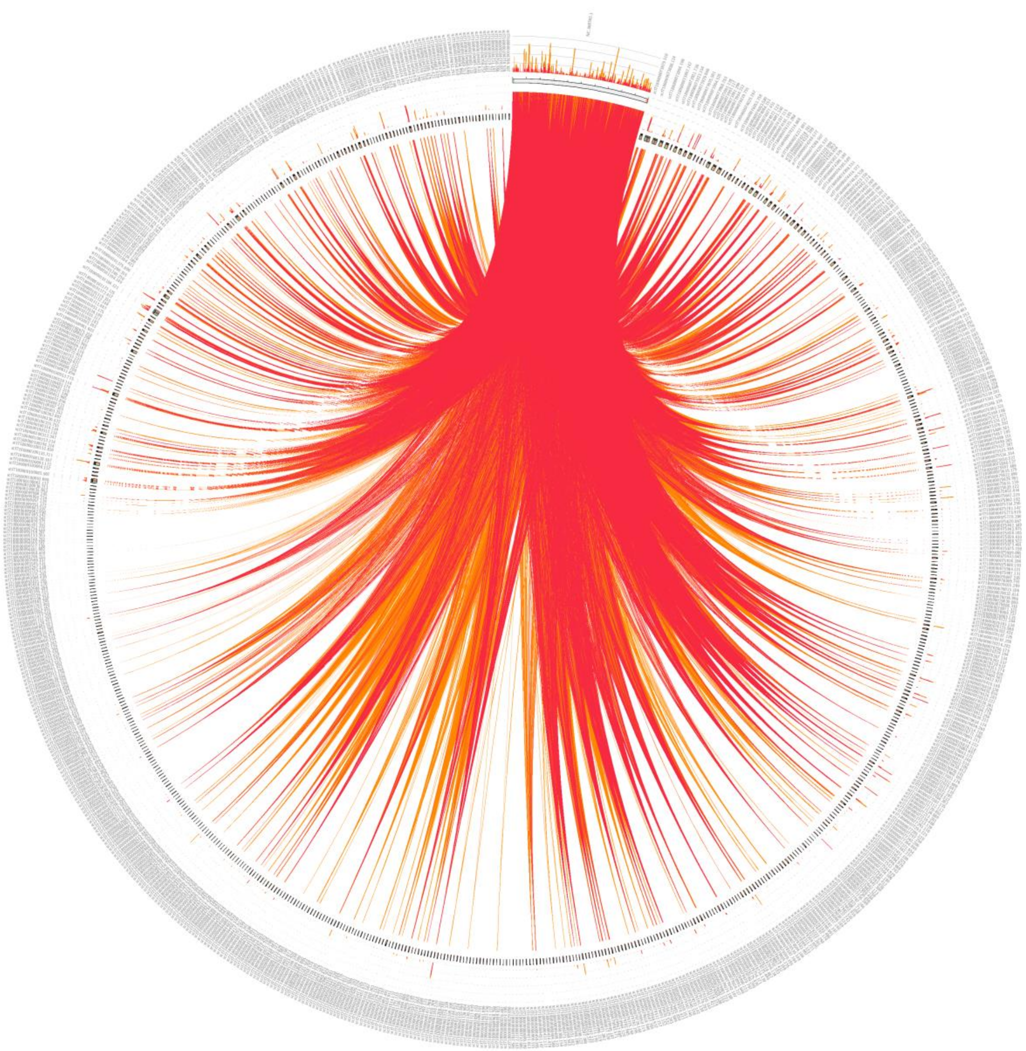

Supplemental Figure S6h Details of microsynteny blocks of assembled contigs of *C. weightii* with chromosome-8 of *C. sinenesis*

Chr-9

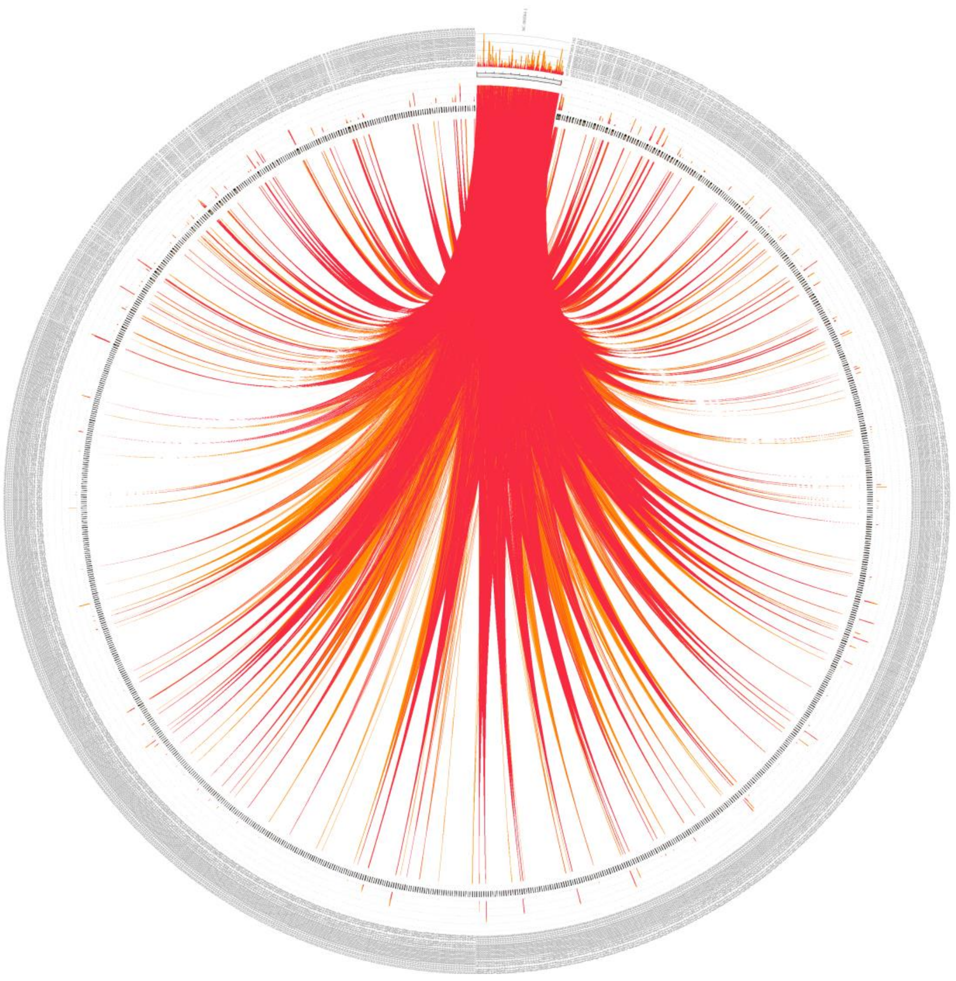

Supplemental Figure S6i Details of microsynteny blocks of assembled contigs of *C. weightii* with chromosome-9 of *C. sinenesis*

cpDNA

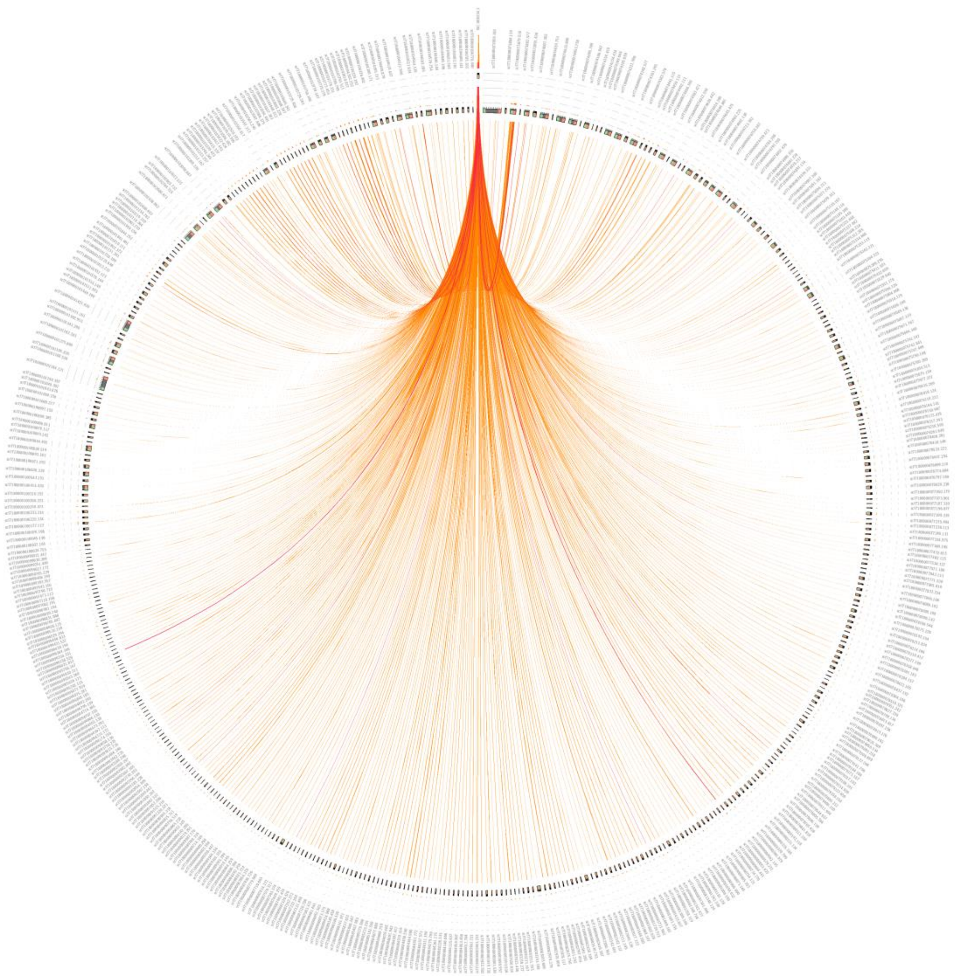

Supplemental Figure S6j Details of microsynteny blocks of assembled contigs of *C. weightii* with cp-genome of *C. sinenesis*

mtDNA

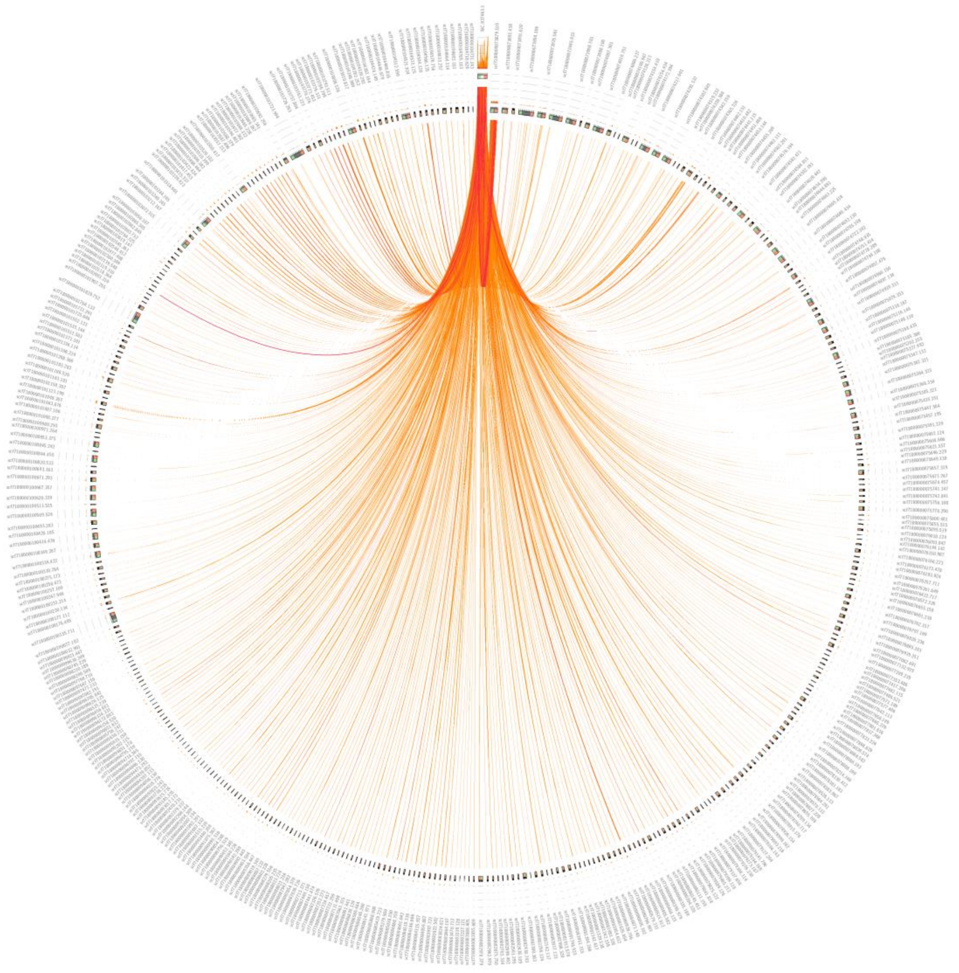

Supplemental Figure S6k Details of microsynteny blocks of assembled contigs of *C. weightii* with mt-genome of *C. sinenesis*
