# Supplementary material for: De Novo Hybrid Assembled Draft Genome of Commiphora wightii (Arnott) Bhandari Reveals Key Enzymes Involved in Phytosterol Biosynthesis
Source: Life (Basel). 2023 Feb 28;13(3):662. doi: 10.3390/life13030662 (PMC10052710; doi:10.3390/life13030662)
Supplement: Supplementary file 1 [file life-13-00662-s001.zip › life-2022038-supplemental Table S1.pdf]

Supplemental Table S1. Details of the repeat analysis performed through RepeatMasker vs 2.2 using *Arabidopsis* as reference.

**Type of transposable elements**	**Number of elements**	**Length occupied (bp)**	**Percentage of sequence (%)**
Retroelements	77277	83991441	8.12
SINEs	0	0	0.00
Penelope	0	0	0.00
LINEs	247	42768	0.00
CRE/SLACS	0	0	0.00
L2/CR1/Rex	0	0	0.00
R1/LOA/Jockey	0	0	0.00
R2/R4/NeSL	0	0	0.00
RTE/Bov-B	0	0	0.00
L1/CIN4	247	42768	0.00
LTR elements	77030	83948673	8.12
BEL/Pao	0	0	0.00
Ty1/Copia	37567	44796163	4.33
Gypsy/DIRS1	37754	38902484	3.76
Retroviral	0	0	0.00
DNA transposons	10765	1920769	0.19
hobo-Activator	2698	607643	0.06
Tcl-IS630-Pogo	9	1254	0.00
En-Spm	0	0	0.00
MuDR-IS905	0	0	0.00
PiggyBac	0	0	0.00
Tourist/Harbinger	392	30985	0.00
Other (Mirage, P-element, Transib	0	0	0.00
Rolling-circles	0	0	0.00
Unclassified	1501	274729	0.03
Total interspersed repeats		86186939	8.33
Small RNA	1504	699663	0.07
Satellites	6	394	0.00
Simple repeats	9555	910378	0.09
