# Supplementary material for: De Novo Hybrid Assembled Draft Genome of Commiphora wightii (Arnott) Bhandari Reveals Key Enzymes Involved in Phytosterol Biosynthesis
Source: Life (Basel). 2023 Feb 28;13(3):662. doi: 10.3390/life13030662 (PMC10052710; doi:10.3390/life13030662)
Supplement: Supplementary file 1 [file life-13-00662-s001.zip › life-2022038-supplemental Table S6.pdf]

Sup Table S6. Total number of 23 plant species selected as reference for constructing the phylogenetic tree.

Sl. No.	Plant species	Order	Family	Genome size (Mb)
1	*Citrus clementiana*	Sapindales	Rutaceae	301.36
2	*Citrus sinensis*	Sapindales	Rutaceae	327.83
3	*Citrus unshiu*	Sapindales	Rutaceae	354.65
4	*Arabidopsis lyrata*	Brassicales	Brassicaceae	206.82
5	*Arabidopsis thaliana*	Brassicales	Brassicaceae	119.66
6	*Brassica cretica*	Brassicales	Brassicaceae	412.52
7	*Brassica nupus*	Brassicales	Brassicaceae	976.19
8	*Brassica oleracea*	Brassicales	Brassicaceae	488.95
9	*Brassica rapa*	Brassicales	Brassicaceae	401.92
10	*Camelina sativa*	Brassicales	Brassicaceae	641.35
11	*Capsella rubella*	Brassicales	Brassicaceae	133.06
12	*Eutrema salsugineum*	Brassicales	Brassicaceae	243.11
13	*Raphanus sativus*	Brassicales	Brassicaceae	426.61
14	*Corchorus capsularis*	Malvales	Malvaceae	317.17
15	*Corchorus olitorius*	Malvales	Malvaceae	334.91
16	*Durio zibethinus*	Malvales	Malvaceae	715.23
17	*Gossypium arboreum*	Malvales	Malvaceae	1694.6
18	*Gossypium hirsutum*	Malvales	Malvaceae	2189.14
19	*Gossypium raimondii*	Malvales	Malvaceae	761.56
20	*Herrania umbratica*	Malvales	Malvaceae	234.039
21	*Theobroma cacao*	Malvales	Malvaceae	324.88
22	*Punica granatum*	Myrtales	Lythraceae	296.38
23	*Eucalyptus grandis*	Myrtales	Myrtaceae	691.43
