# Supplementary material for: De Novo Hybrid Assembled Draft Genome of Commiphora wightii (Arnott) Bhandari Reveals Key Enzymes Involved in Phytosterol Biosynthesis
Source: Life (Basel). 2023 Feb 28;13(3):662. doi: 10.3390/life13030662 (PMC10052710; doi:10.3390/life13030662)
Supplement: Supplementary file 1 [file life-13-00662-s001.zip › life-2022038-supplemental Table S8.pdf]

Supplemental Table S8. Result showing the total number of SSRs found in the draft genome of *Commiphora wightii* along with their distribution into different classes.

**Parameters**	**Measure**
Total number of sequences examined	22, 822
Total size of examined sequences (bp)	1,034,226,503
Total number of identified SSRs including mono, di, tri, tetra, penta and hexa nucleotide repeats	3,86,735 (mono-274858, di-80992, tri-24606, tetra-3982, penta-1724, hexa-573)
Number of SSR containing sequences	22,294
Number of sequences containing more than one SSR	20,881
Number of SSRs present in simple formation	3,38,755
Number of SSRs present in compound formation	47,980
